# Supplementary material for: Long-chain anionic surfactants enabling stable perovskite/silicon tandems with greatly suppressed stress corrosion
Source: Nat Commun. 2023 Apr 15;14:2166. doi: 10.1038/s41467-023-37877-z (PMC10105702; doi:10.1038/s41467-023-37877-z)
Supplement: Supplementary file 1 — Supplementary Information [file 41467_2023_37877_MOESM1_ESM.pdf]

Supplementary Materials for

Long-chain anionic surfactants enabling stable perovskite/silicon tandems with greatly suppressed stress corrosion

Xinlong Wang[1,2], Zhiqin Ying[1*], Jingming Zheng[1], Xin Li[1], Zhipeng Zhang[3], Chuanxiao Xiao[1,4], Ying Chen[1], Ming Wu[1], Zhenhai Yang[1], Jingsong Sun[1], Jia-Ru Xu[5], Jiang Sheng[1], Yuheng Zeng[1], Xi Yang[1*], Guichuan Xing [3], Jichun Ye[1*]

[1]Ningbo Institute of Materials Technology and Engineering, Chinese Academy of Sciences (CAS), Ningbo 315201, China

[2]University of Chinese Academy of Sciences, No.19(A) Yuquan Road, Shijingshan District, Beijing 100049, China

[3]Institute of Applied Physics and Materials Engineering, University of Macau, Macao SAR, 999078 China

[4]Ningbo New Materials Testing and Evaluation Center CO., Ltd. Ningbo City, Zhejiang Province, 315201, China

[5]Celanese (China) Holding Co., Ltd. Asia Technology and Innovation Center, Shanghai 201210, China

*Correspondence: yingzhiqin@nimte.ac.cn (Z.Y.), yangx@nimte.ac.cn (X.Y.), jichun.ye@nimte.ac.cn (J.Y.)

This Supplementary Materials includes:

Supplementary Notes 1 – 7,

Supplementary Figs. 1 – 56,

Supplementary Tables 1 – 4,

Supplementary references.

Supplementary Note 1

Revealing the effects of mechanical properties on the stress of perovskite films.

The stress in the perovskite film mainly originates from the differences in the relative volume change of the perovskite and the underlying substrate after removal from the processing conditions, i.e, annealing (often above 100 °C) and cooling processes. The CTE can be extracted from the slope via the formula of $\Delta d/d$ = CTE×ΔT by plotting the lattice constant changes ($\Delta d/d$) as a function of temperature variation ($\Delta T = T - 298$ K) (Supplementary Fig. 4), then the residual stress values of the perovskite films annealed at different temperatures can be determined based on the measured YM and CTE[1]. This residual stress is typically dependent on the fundamental mechanical parameters of both substrate and perovskite materials, and can be given by

$$\sigma_{\Delta T} = \frac{E_P}{1\text{-}v_P}(\alpha_S - \alpha_P)\Delta T \quad (1)$$

where E_P and v_P refer to YM and Poisson's ratio in the perovskite (v_P = 0.3), respectively, while α_S and α_P represent the CTE of substrate and perovskite, respectively, and ΔT is the temperature gradient during the perovskite fabrication. Although numerous studies provide valuable guidance for the stain engineering of perovskite, most of them have focused on examining the impact of ΔT or α_S on the stress evolution, such as reducing the annealing temperature[1], using plastic substrates with α_S that are similar to α_P (ref. [2]), and inserting a compliant interlayer to minimize the mismatch between the α_S and the α_P at the interface[3]. The effect of the mechanical property of the perovskite material itself, especially the variation of E_P and the α_P in the perovskite film, has been largely ignored.

Supplementary Note 2

Grazing-incidence X-ray diffraction (GIXRD) technique with the 2*θ*–sin²ψ method.

We estimate the stresses of different perovskite films by the grazing-incidence X-ray diffraction (GIXRD) technique with the 2θ–$\sin^2\psi$ method (Supplementary Fig. 5)[4]. Generally, the residual stress can be reflected by the slope of linear fitted 2θ–$\sin^2\psi$ and the perovskite film stress (σ) can be calculated according to the following equation:

$$\sigma = -\frac{E}{2(1+v)}\frac{\pi}{180}\cot\theta_0\frac{\partial(2\theta)}{\partial(\sin^2\psi)} \quad (2)$$

where E and v are Young's modulus and Poisson's ratio for perovskite films, respectively. θ_0 is the diffraction peak for a stress-free perovskite crystal plane. θ is the diffraction peak for the measured perovskite thin films. The (012) plane is chosen for further analysis due to its high diffraction angle and multiplicative factor, providing the reliable alleviate orientation effect and structure symmetry information. ψ is the angle of the diffraction vector with respect to the sample normal direction. The 2θ values are linked with the varied instrument tilt angle ψ of 0°, 10°, 20°, 30°, 40° and 50° to obtain the corresponding XRD curves, as shown in Supplementary Fig. 5a–c. The slopes of the fitting lines for the control, $[C_4mim]^+[C_0SO_4]^-$ treated and $[C_4mim]^+[C_8SO_4]^-$ treated films have negative values, indicating all the samples are subjected to tensile stress. Compared with the slope of the fitting line for the control film (–3.8%), $[C_4mim]^+[C_0SO_4]^-$ treated film shows a larger slope in the absolute value of –5.2%, implying the increasing residual tensile stress in $[C_4mim]^+[C_8SO_4]^-$ treated

film. However, in the $[C_4mim]^+[C_8SO_4]^-$ treated film, the fitting line shows a much smaller slope of –1.4% in absolute value, implying the significantly decreased residual tensile stress in $[C_4mim]^+[C_8SO_4]^-$ treated film. According to equation (2), the residual stresses of control, $[C_4mim]^+[C_0SO_4]^-$ treated and $[C_4mim]^+[C_8SO_4]^-$ treated films are 15.5 MPa, 25.4 MPa and 4.6 MPa, respectively (Supplementary Fig. 5d).

Supplementary Note 3

The analysis of residual stress in the perovskite films from Raman spectroscopy measurements.

To verify the decreased stress induced by the $[C_4mim]^+[C_8SO_4]^-$ additive, we perform Raman spectroscopy on the fresh-fabricated perovskite films with and without $[C_4mim]^+[C_8SO_4]^-$. The perovskite powder prepared by scraping the as-prepared perovskite films from substrates is used as the stress-free reference. Since the perovskite is not Raman-active at room temperature, we utilize the laser-induced decomposition product PbI_x to reflect the local stress in the perovskite thin film, considering the interaction between PbI_x and the adjacent perovskite[5]. The stress evolution can then be estimated by using the information obtained from the shift of vibrational modes in the Raman spectra[6]. Two peaks are appearing at 78.6/139.2 cm^{-1}, 76.4/133.8 cm^{-1} and 78.6/139.2 cm^{-1} for the perovskite powder, perovskite film without and with $[C_4mim]^+[C_8SO_4]^-$, respectively (Supplementary Fig. 6a). Compared with the scraped powder, the A_{1g} Raman spectrum of PbI_x for the control sample is shifted to lower wavenumbers. We attribute this redshift to the larger interlayer spacing, thereby indicating the strong tensile stress along the out-of-plane direction. Instead, upon introducing $[C_4mim]^+[C_8SO_4]^-$ into the perovskite film, almost no Raman peak shift can be observed, a clear indicator of the decreased tensile stress in the perovskite film. The evolution of the Raman peak with increasing illumination times is also characterized to further validate the stress differences. The integration time is 10s for each test and the time interval in between is 0.1 s. The results are consistent: the control film presents distinguishable peak broadening in Raman patterns (Supplementary Fig. 6b), accompanied by a large Raman peak shift from 133.8 to 139.2 cm^{-1}, indicating the relaxation of the film residue stress during the illumination, however, no distinct variation of Raman peak can be observed for the $[C_4mim]^+[C_8SO_4]^-$ based film and perovskite powder (Supplementary Fig. 6c,d), confirming the reduced film residue stress induced by the $[C_4mim]^+[C_8SO_4]^-$.

Supplementary Note 4

The role of the additive segregation on the perovskite nucleation and mechanical engineering.

For the control sample (Supplementary Fig. 10), it is well known that the initial perovskite heterogeneous nucleation, which is triggered by the supersaturation of precursor solution after anti-solvent dripping, randomly occurs at the solution surface due to the rapid solvent evaporation. Although each nucleus serves as a template for further crystallization and undergoes subsequent growth to form a larger crystalline grain, the crystal orientation is gradually disordered due to the isotropic environment[7], thus resulting in a perovskite film with a random orientation. After the introduction of the $[C_4mim]^+[C_0SO_4]^-$ additive (Supplementary Fig. 11), the terminal $–SO_4$ groups

serve as additional heterogeneous nucleation sites. Due to the additive downward segregation as mentioned above, the nucleation simultaneously occurs at both the top air/solution and bottom solution/substrate interfaces, i.e., throughout the bulk solution. Such high-density nuclei largely confine the subsequent perovskite growth to a limited space at each nucleation site, thus detrimentally reducing the perovskite grain size.

Supplementary Note 5

Preferential orientation and morphology analysis by GIWAXS measurements.

To further explore the influence of stress engineering on the perovskite crystal orientation, we conduct the GIWAXS measurements for the control and $[C_4mim]^+[C_8SO_4]^-$ treated perovskite films (Supplementary Fig. 16). The GIWAXS patterns of the $[C_4mim]^+[C_8SO_4]^-$ treated sample show higher intensities along the (001) ring (|q| = 0.95−1.08 Å^{-1}) and with the increased peak intensity ratios of (001)/(011) and (001)/(111) in both in-plane and out-of-plane directions, respectively, indicating a better crystallinity compared to that of the control sample and suggesting that the stress relaxation can indeed reduce the crystal misorientation[8,9]. This can be further supported by the corresponding azimuthal scan profiles for the (001) lattice plane (along the ring at q = 1.0 Å^{-1}) extracted from the 2D-GIWAXS patterns, where the $[C_4mim]^+[C_8SO_4]^-$ treated sample exhibits an enhanced intensity of (001) lattice plane diffraction peak at azimuth angles in the range of 20°−60° but a decreased peak intensity at azimuth angles of 60°−120° in compared with the control sample. Additionally, the diffraction peaks at q approximate to 0.9 Å^{-1} corresponding to PbI_2 are notably suppressed in the $[C_4mim]^+[C_8SO_4]^-$ treated sample, suggesting the significant suppression of the stress-induced perovskite decomposition. The enlarged grain size of the perovskite film can also be confirmed by the scanning electron microscopy (SEM) results (Supplementary Fig. 18) where the grain size is increased from ~180 nm to ~340 nm.

Supplementary Note 6

Tests of trap density by pump fluence dependent PL.

To investigate the effect of stress on trap density in perovskite films, we obtain pump fluence dependent PL measurements for purpose of qualifying the concentration of trap states in control and target films. Under low fluence fs laser pulse excitation (where Auger recombination is negligible) and the assumption that trap states recombination is much slower than band edge radiative recombination, the increase in PL intensity is slow when the pump fluence energy is elevated, because a portion of photogenerated carriers is captured by traps. But once the pump fluence energy is higher than a threshold, traps can be completely filled, which leads to a much more dramatic increase in PL intensity with an increase in the pump fluence energy. Based on this, the photogenerated charge carrier density $n_C(0)$ after photoexcitation can be described with a set of differential equations and is given by:

$$n_C(0)=\sum_i n^i_{TP}(0)\left(1-\frac{e^{-\alpha_i\tau_0}I_{PL}}{k}\right)+\frac{I_{PL}}{k} \quad (3)$$

where I_{PL} is the integrated PL intensity which relates to the carrier density by $I_{PL}=k\int_0^\infty n_C(t)/\tau_0 d(t)$, where k is constant for a given sample, τ_0 is the PL lifetime, $n^i_{TP}(0)$ is the initial unfilled trap state density, and a_i is the product of the trap cross section and the carrier velocity (a parameter unique

for each type of trap). The summation over i takes into account different types of traps in the perovskite film contains a significant amount of both surface and bulk traps. According to equation (3), the trap density can thus be determined by fitting the n_C vs. I_{PL}/k curve. Fig. 3c displays the photogenerated carrier density vs. the integrated PL intensity for control films and target films, and the fitting parameters are listed in Supplementary Table 3. Two types of traps are revealed in the model, with the bulk (surface) traps exhibiting fast (slow) trapping times.

Supplementary Note 7

Electric poling experiment.

The electric poling experiment is utilized to investigate the ions migration property of perovskite[10,11]. With the structure of ITO/Au/perovskite/Au, the thickness of the bottom electrode Au is around 10 nm for light transparency. The Au top electrode with a thickness of 200 nm is deposited under the patterned mask with 0.1 cm^2 (Supplementary Fig. 20). The unpolarized devices exhibit ohmic resistance under 1-sun illumination (Fig. 3f) as expected. And the devices also show zero J_{SC} and V_{OC} because the electrodes, made with the same material (Au), do not induce preferential directionality (asymmetry) for charge transport. With a poling bias applied on the two electrodes, regions with *p*-type doping by negatively charged ions vacancies and *n*-type doping by positively charged ions vacancies are created next to the positive and negative electrodes, respectively, resulting in a built-in potential (V_{bi}) of poling films (Fig. 3f). A V_{OC} can be detected to reflect the V_{bi} by illuminated the poled devices. The magnitude of V_{OC} is essentially determined by the number of polarized ions toward two sides. Specifically, the more ions piled up at the electrodes, the higher the V_{oc}.

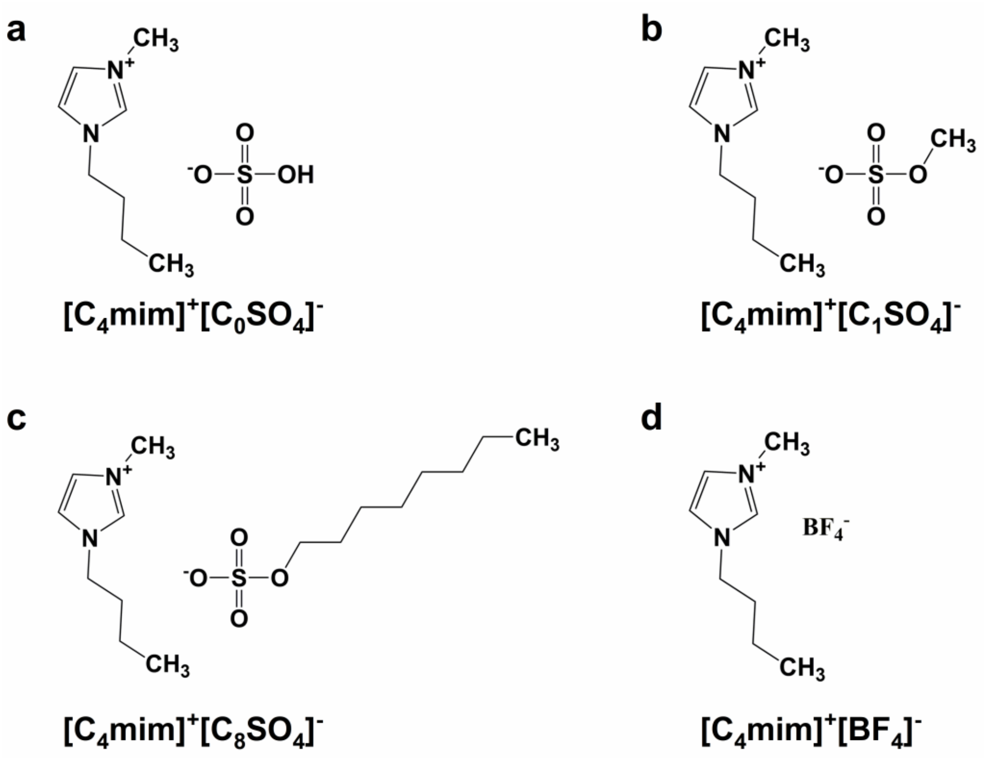

Supplementary Figure 1. Chemical structures of different additives. The corresponding chemical structures of (**a**) 1-Butyl-3-methylimidazolium hydrogen sulfate ($[C_4mim]^+[C_0SO_4]^-$), (**b**) 1-Butyl-3-methylimidazolium methyl sulfate ($[C_4mim]^+[C_1SO_4]^-$), (**c**) 1-Butyl-3-methylimidazolium octyl sulfate ($[C_4mim]^+[C_8SO_4]^-$) and (**d**) 1-Butyl-3-methylimidazolium tetrafluoroborate ($[C_4mim]^+[BF_4]^-$).

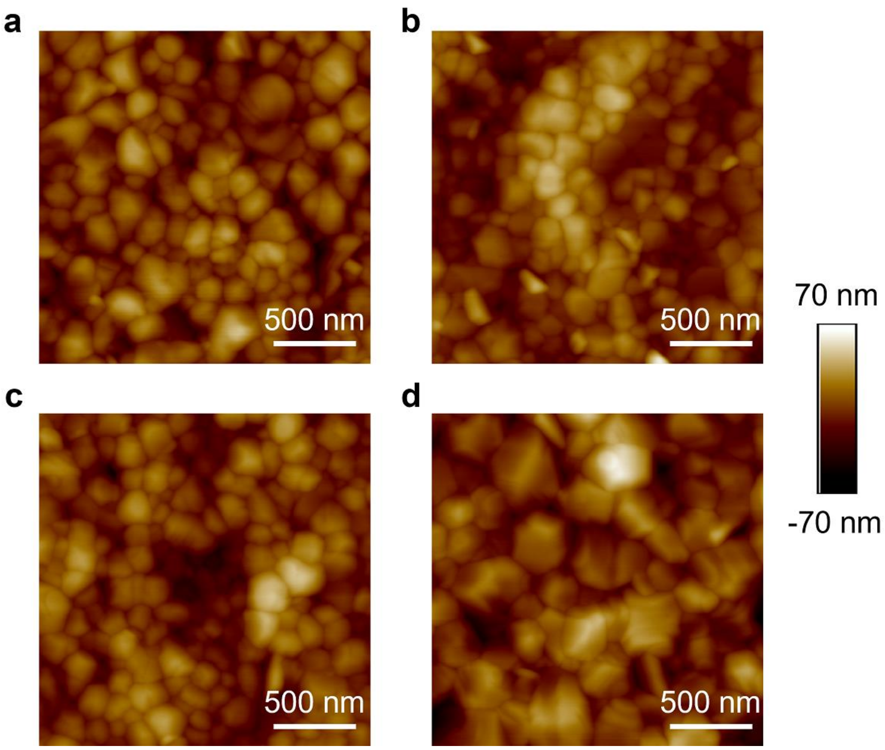

Supplementary Figure 2. Topography of perovskite films. AFM topography mapping of perovskite thin films without (referred as control sample) (a) additives, $[C_4mim]^+[C_0SO_4]^-$ (b), $[C_4mim]^+[C_1SO_4]^-$ (c) and $[C_4mim]^+[C_8SO_4]^-$ (d) treated films. Compared with the control film, the perovskite films exhibit a slightly decreased grain size after the incorporation of $[C_4mim]^+[C_0SO_4]^-$ and $[C_4mim]^+[C_1SO_4]^-$. On the contrary, the grains became larger for the $[C_4mim]^+[C_8SO_4]^-$–based perovskite film.

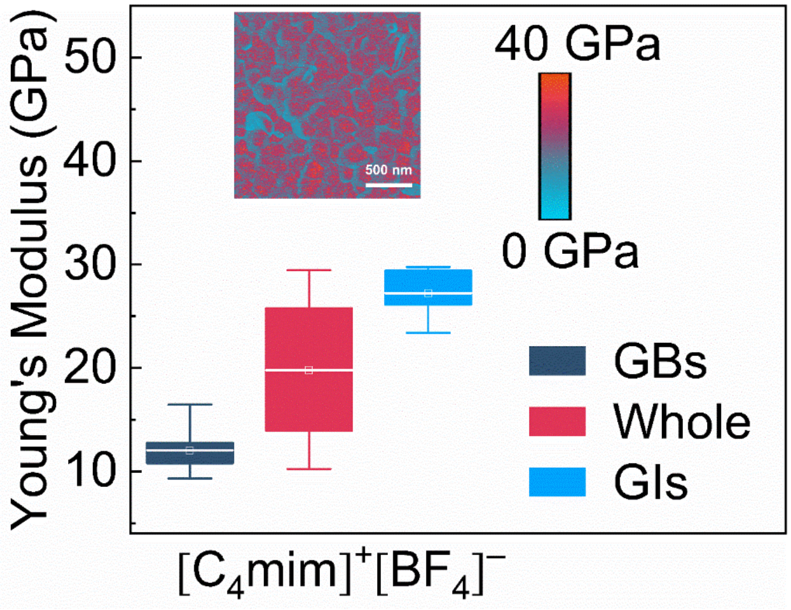

Supplementary Figure 3. Modulus mapping of $[C_4mim]^+[BF_4]^-$ treated perovskite films. PFQNM-AFM characterization of $[C_4mim]^+[BF_4]^-$ treated perovskite films (modulus map is shown in the inset). The color scale bar represents the magnitude of modules. The box plot denotes the YM values across the whole images (red) and separately for the regions identified as GBs (navy) or as GIs (blue).

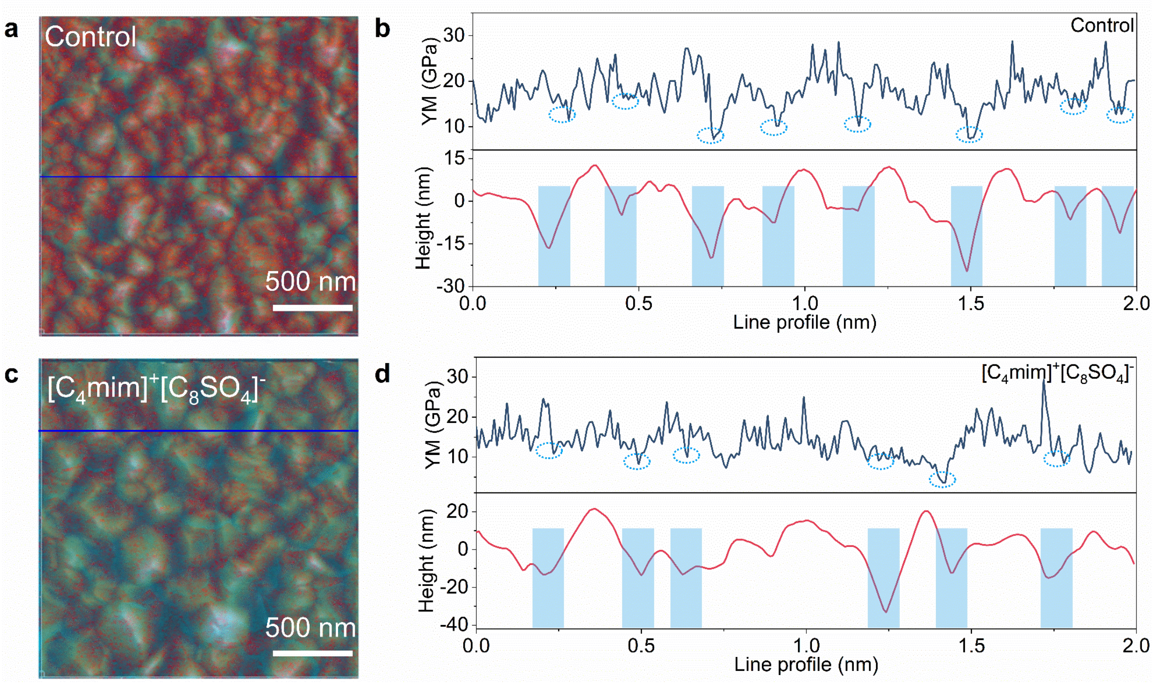

Supplementary Figure 4. Overlay of the topography and YM maps with the relative height and YM line-scan spectra. (**a,c**) Overlay of the topography and YM maps, revealing the existence of subdomains of different stiffness within individual morphological grains. The red domain with a high average YM can be attributed to the contribution of grain intragranular regions, whereas the blue domain with a low average YM belongs to the region near grain boundaries. (**b,d**) The relative height and YM line-scan spectra of the perovskite films (**b**) without additives (referred as control sample) and (**d**) $[C_4mim]^+[C_8SO_4]^-$ treated perovskite films extracted from the corresponding maps, and the grain boundaries were marked with blue dotted circles and the blue shadows.

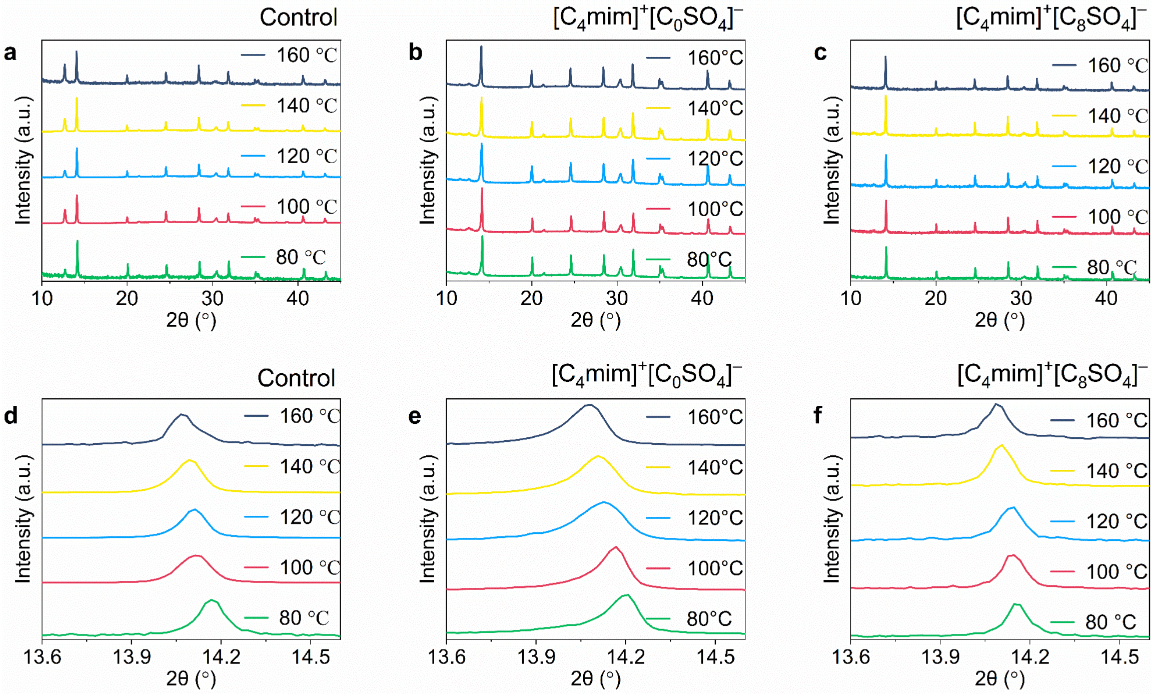

Supplementary Figure 5. The XRD patterns of perovskite films fabricated at different temperatures. The XRD patterns of (**a**) control, (**b**) $[C_4mim]^+[C_0SO_4]^-$ and (**c**) $[C_4mim]^+[C_8SO_4]^-$ treated films fabricated at different temperatures, and magnified (001) diffraction peaks of (**d**) control, (**e**) $[C_4mim]^+[C_0SO_4]^-$ treated and (**f**) $[C_4mim]^+[C_8SO_4]^-$ treated films. All samples show XRD peaks (2θ) at around 14.1°, 20.0°, and 24.5°, corresponding to (001), (011), and (111) crystallographic planes, respectively. Since the most reliable structural symmetry information can be provided by a high diffraction angle with a multiplication factor, the crystal plane (001), among the three planes with stronger diffraction peaks, was selected for further XRD analysis.

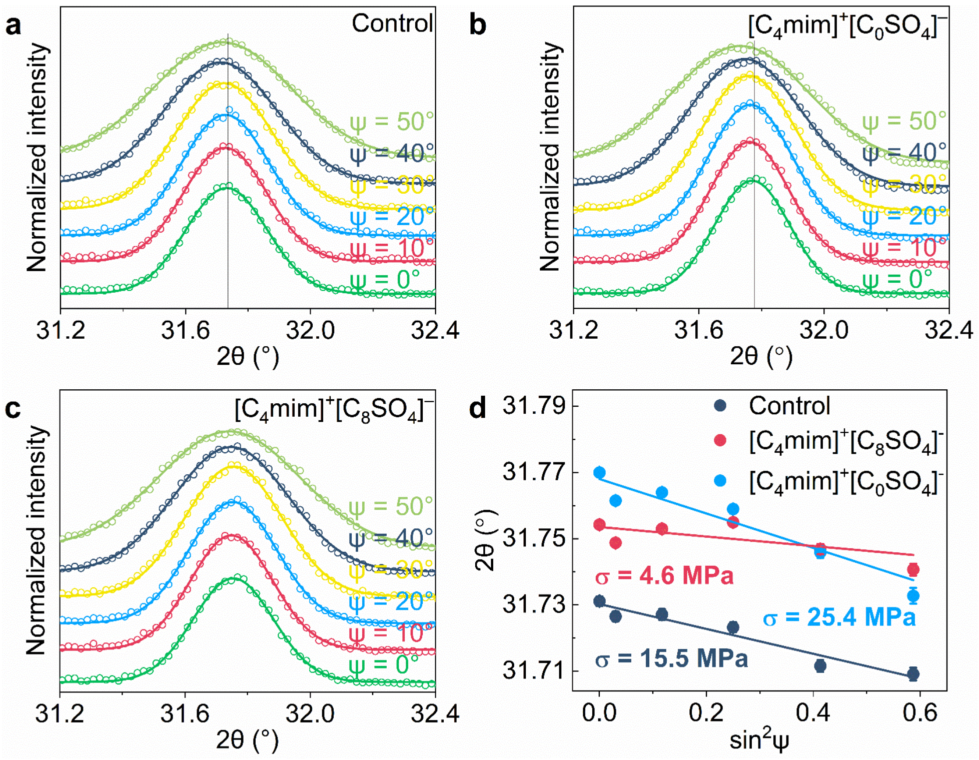

Supplementary Figure 6. The residual stresses in the perovskite films. GIXRD spectrum at different tilt angles for the (**a**) control, (**b**) $[C_4mim]^+[C_0SO_4]^-$ and (**c**) $[C_4mim]^+[C_8SO_4]^-$ treated films. (**d**) Residual stresses of control, $[C_4mim]^+[C_0SO_4]^-$ treated and $[C_4mim]^+[C_8SO_4]^-$ treated films (measured (points) and Gauss fitted (lines) diffraction stress data as a function of $\sin^2\psi$). The error bar indicates standard deviation of the 2θ.

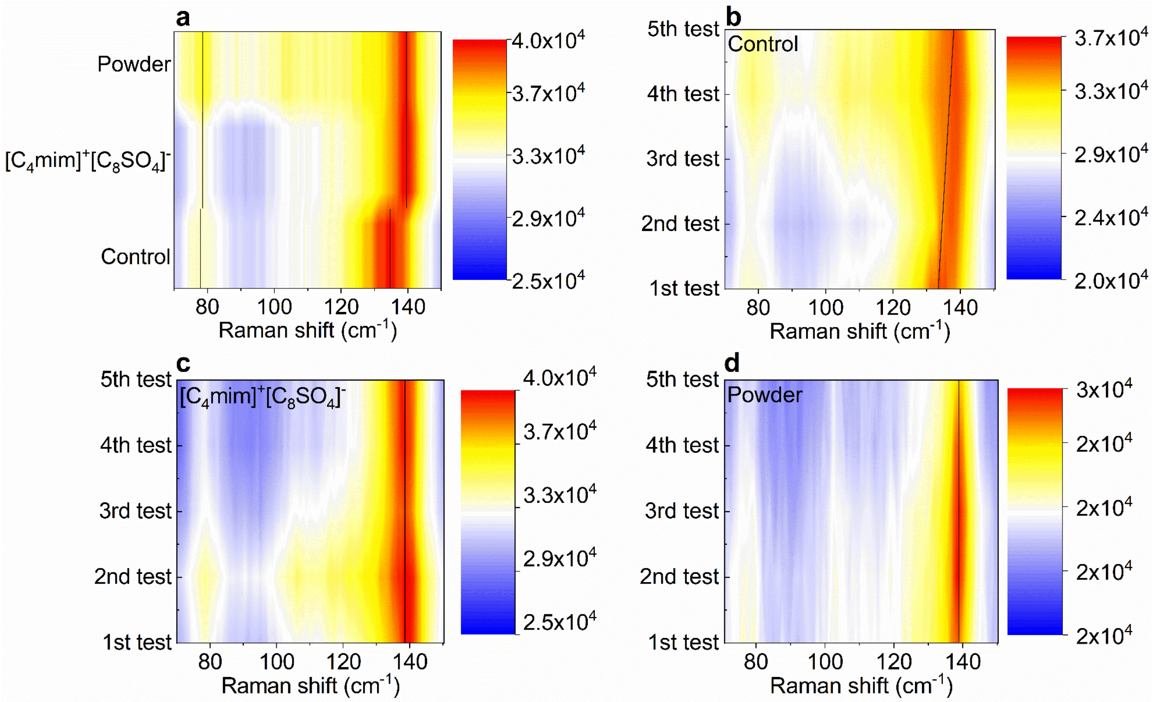

Supplementary Figure 7. The analysis of residual stress in the perovskite films. (**a**) Raman mapping of fresh-fabricated control film, $[C_4mim]^+[C_8SO_4]^-$ treated film and perovskite powder. The evolution of the Raman peaks for the (**b**) control film, (**c**) $[C_4mim]^+[C_8SO_4]^-$ treated film and (**d**) perovskite powder. The integration time is 10s for each test and the time interval in between is 0.1 s. The colored scale bar represents the magnitude of Raman intensity.

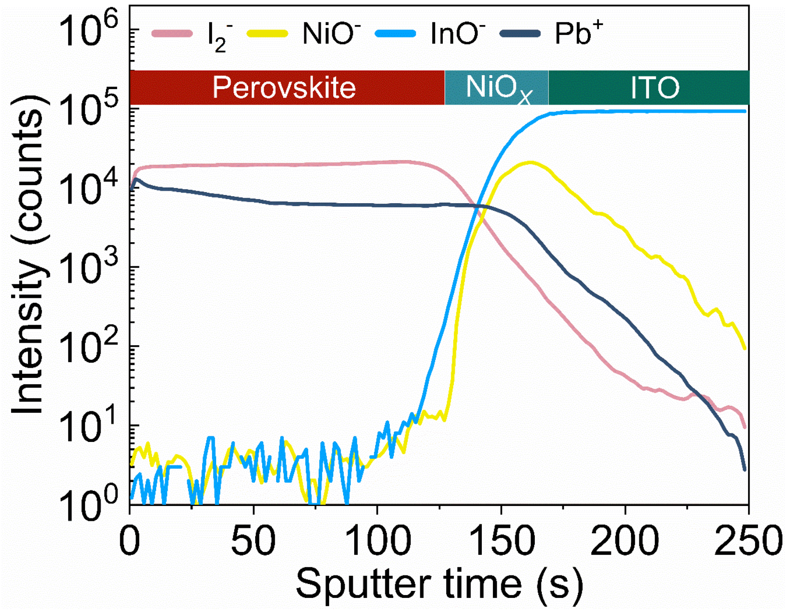

Supplementary Figure 8. ToF-SIMS measurements. ToF-SIMS depth profiles of the control perovskite films.

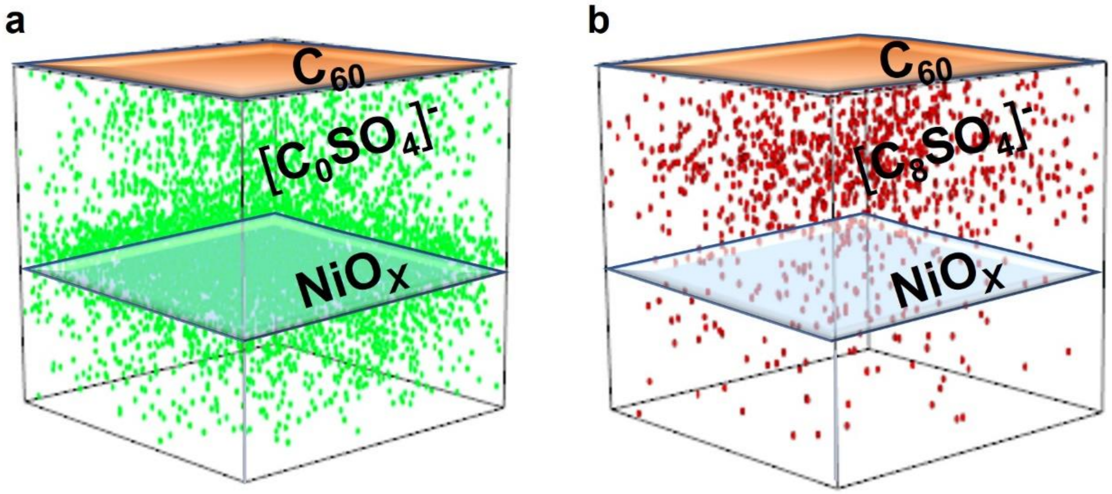

Supplementary Figure 9. The reconstructed 3D images of (a) $[C_0SO_4]^-$ and (b) $[C_8SO_4]^-$ in the $[C_4mim]^+[C_0SO_4]^-$ treated and $[C_4mim]^+[C_8SO_4]^-$ treated perovskite film, respectively. The 3D ToF-SIMS tomography is realized by combining the depth profiling with high-resolution imaging (each 3D reconstruction is 50 × 50 × 0.5 μm).

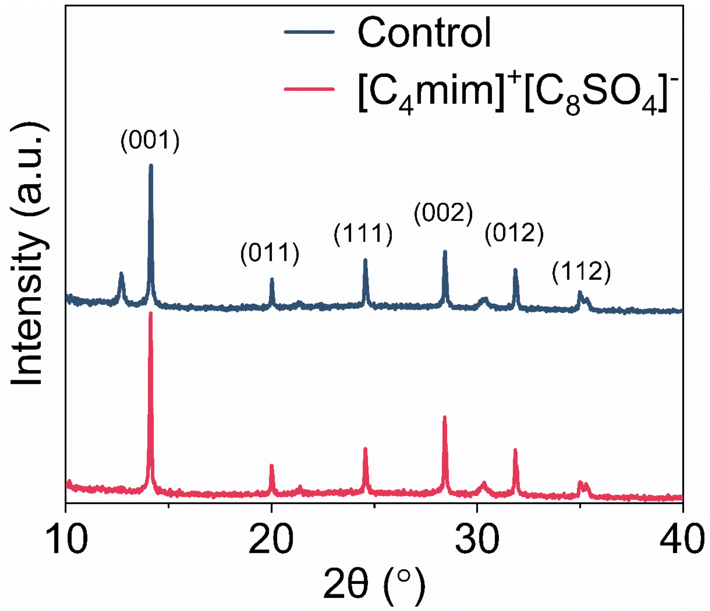

Supplementary Figure 10. The XRD pattern of the control and $[C_4mim]^+[C_8SO_4]^-$ treated films. $[C_4mim]^+[C_8SO_4]^-$ tends to locate at the perovskite grain boundary rather than to substitute into the crystal lattice since the unaltered peak position can be seen in the XRD pattern.

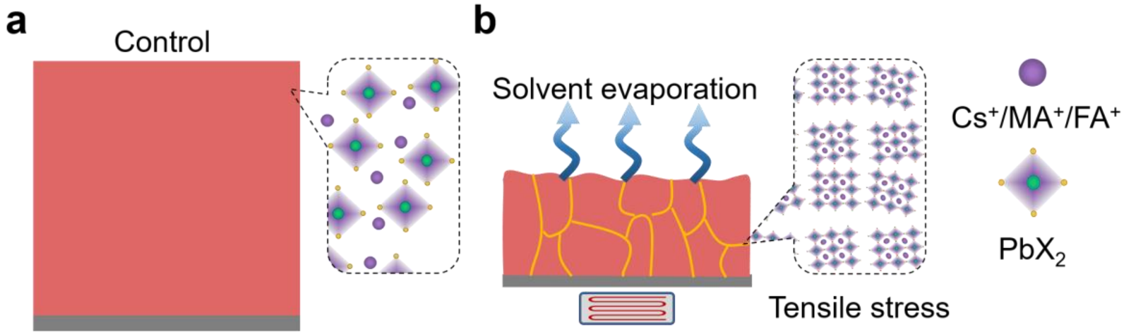

Supplementary Figure 11. The schematic for the mechanism of control film formation. The schematic for the (**a**) spatial distribution and nucleation of perovskite precursor, and (**b**) tensile stress in perovskite film.

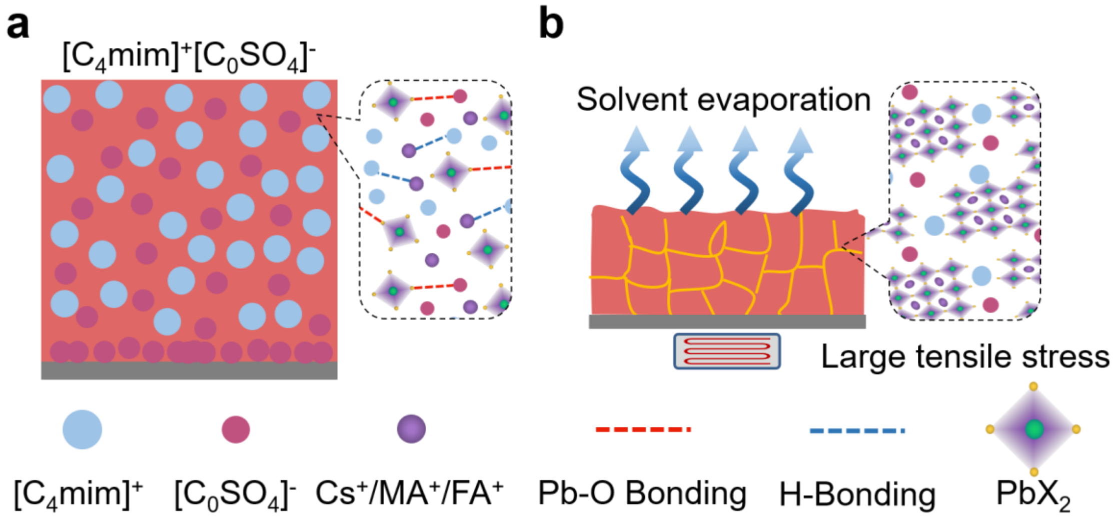

Supplementary Figure 12. The schematic for the mechanism of $[C_4mim]^+[C_0SO_4]^-$ segregation during the film formation. The schematic for the (**a**) spatial distributions of $[C_4mim]^+[C_0SO_4]^-$ additives in perovskite precursor and the role of the additive segregation in the perovskite nucleation and (**b**) mechanical engineering.

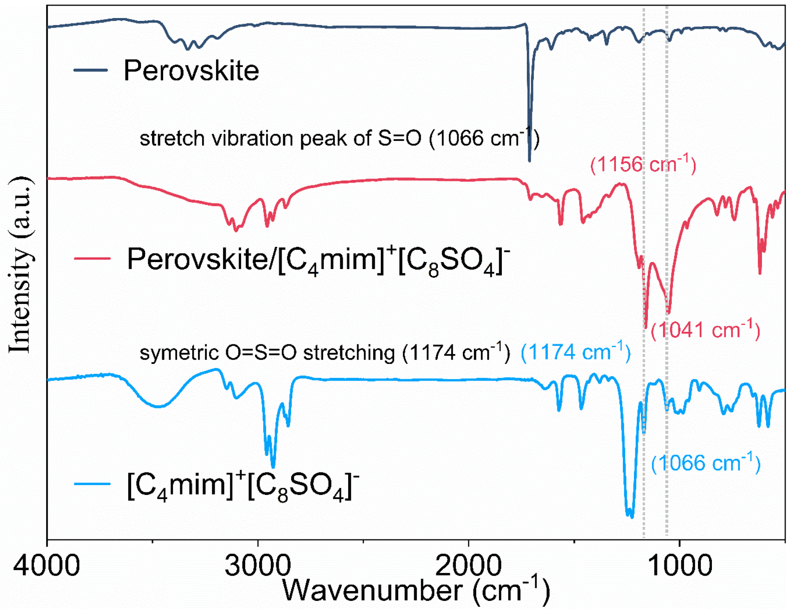

Supplementary Figure 13. FTIR spectra of $[C_4mim]^+[C_8SO_4]^-$, perovskite and a mixture of $[C_4mim]^+[C_8SO_4]^-$ with perovskite. The grey dotted line indicates the stretch vibration of S=O and symmetric stretching of O=S=O, respectively. The interaction can be proved by FTIR spectroscopy measurements, where both stretching vibration of S=O bonds and symmetric O=S=O bonds in the perovskite with $[C_4mim]^+[C_8SO_4]^-$ shift to lower wavenumbers (from ca. 1066 cm^{-1} to ca. 1041 cm^{-1} for S=O, from ca. 1174 cm^{-1} to ca. 1156 cm^{-1} for O=S=O)[12,13], as compared with those in pure $[C_4mim]^+[C_8SO_4]^-$. This shift is also indicative of the formation of an intermediate $[C_8SO_4]^-$–PbI_2 adduct, which is expected to retard the crystal growth and improve the perovskite crystallinity.

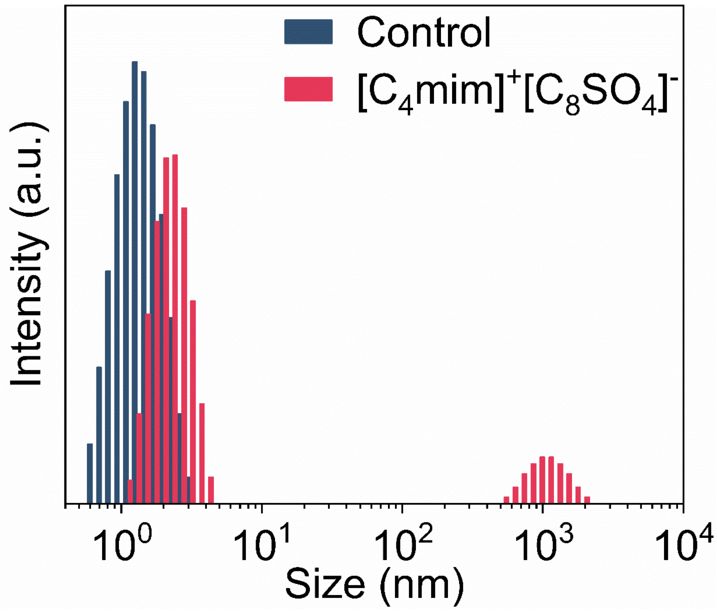

Supplementary Figure 14. Dynamic light scattering (DLS) measurements. DLS of control and $[C_4mim]^+[C_8SO_4]^-$ treated perovskite precursors.

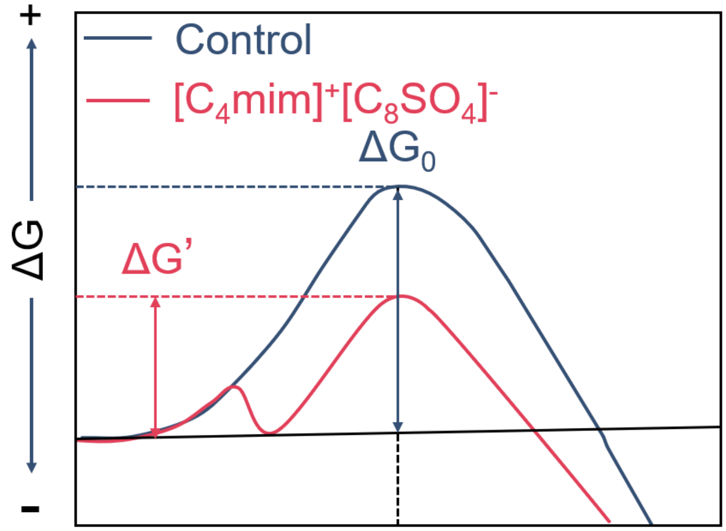

Supplementary Figure 15. Schematic diagram of Gibbs free energy distribution of classical nucleation theory and non-classical nucleation theory. ΔG_0 and ΔG' are the critical Gibbs free energy for classical nucleation and non-classical nucleation, respectively. The addition of $[C_4mim]^+[C_8SO_4]^-$ lowers the energy barrier for forming the pre-nucleated cluster.

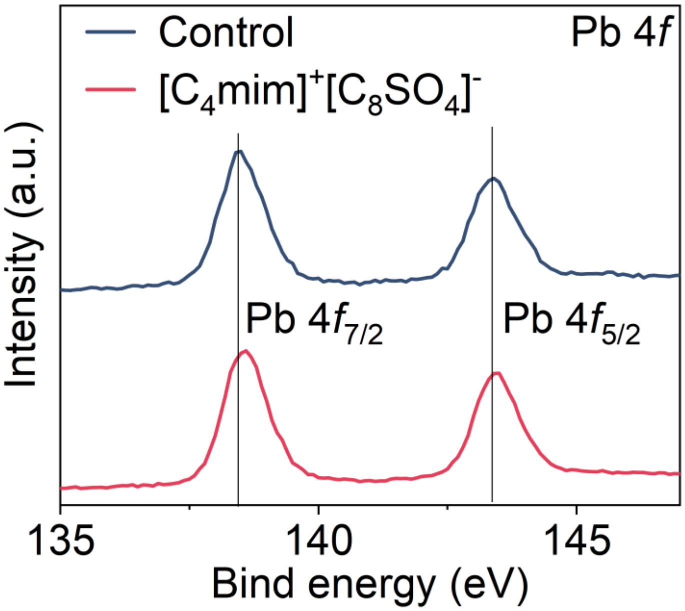

Supplementary Figure 16. XPS spectra control and $[C_4mim]^+[C_8SO_4]^-$ treated perovskite films. High-resolution Pb 4*f* spectra of the control and $[C_4mim]^+[C_8SO_4]^-$ treated films. The vertical lines demarcate the peak positions for the control film. The interaction between $[C_4mim]^+[C_8SO_4]^-$ and perovskite is examined by the XPS. Notably, the binding energy peaks of Pb 4*f* obviously shifted to lower positions after the $[C_4mim]^+[C_8SO_4]^-$ incorporation, which can be attributed to the formation of strong bonding between the Pb atoms in perovskite and O atom in $[C_8SO_4]^-$ based on Lewis acid-base interaction as a consequence of the higher electronegativity of O atoms compared to I atoms[14].

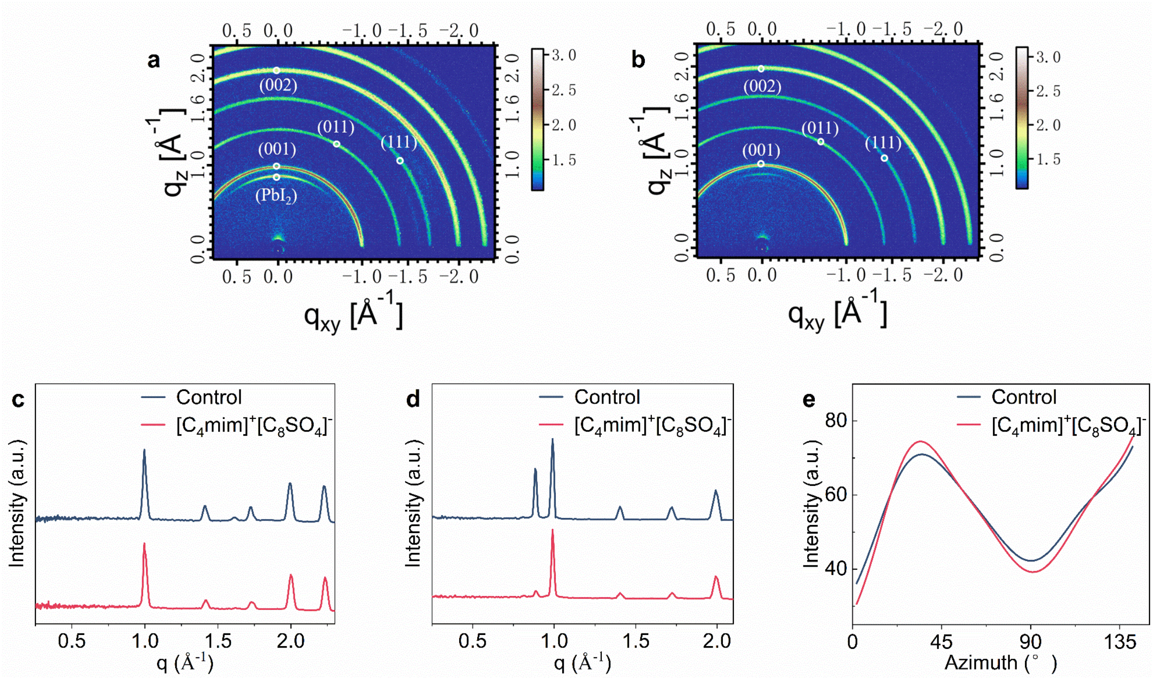

Supplementary Figure 17. Preferential orientation analysis of control and $[C_4mim]^+[C_8SO_4]^-$ treated perovskite films. GIWAXS patterns for (**a**) control and (**b**) $[C_4mim]^+[C_8SO_4]^-$ treated films. The indexed facets are marked with white circles and labeled. The color scale bar represents the diffraction intensity. The (**c**) in-plane and (**d**) out-of-plane scattering profiles for the corresponding samples. (**e**) Integrated intensity plots azimuthally along the ring at *q* approximate to 1 Å^{-1}, assigned to the (001) plane of corresponding perovskite films.

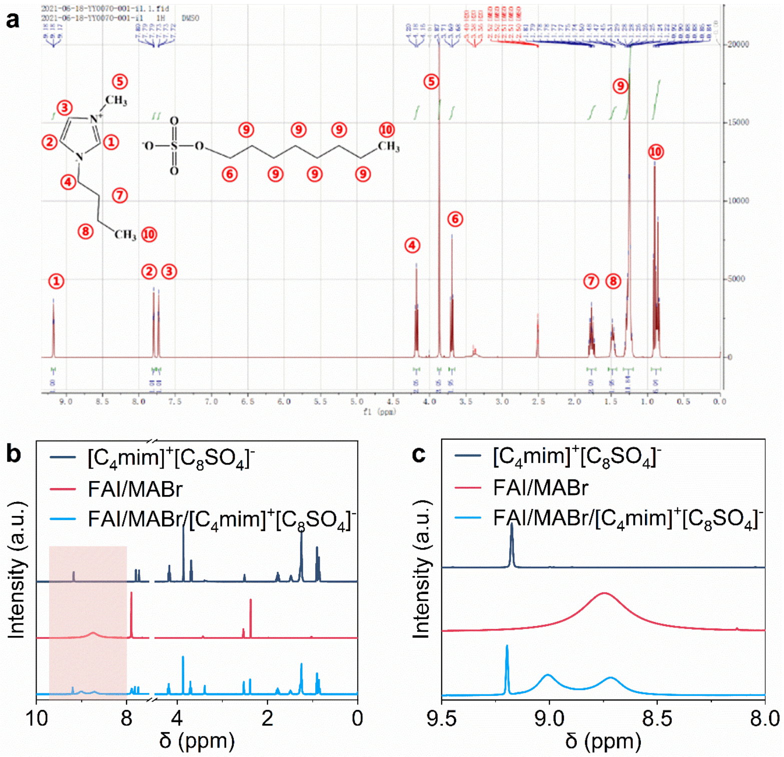

Supplementary Figure 18. ^{1}H–NMR spectra of pure $[C_4mim]^+[C_8SO_4]^-$ solution, the mixed solution of FAI and MABr and the mixed solution of $[C_4mim]^+[C_8SO_4]^-$, FAI and MABr. (**a**) Numbers 1–10 are used to label hydrogens with different environments. (**b**) A comparison of ^{1}H–NMR spectra of $[C_4mim]^+[C_8SO_4]^-$ solution, the mixed solution of FAI and MABr and the mixed solution of $[C_4mim]^+[C_8SO_4]^-$, FAI and MABr. (**c**) Enlarged image of the shaded part of (**b**). A distinct peak shift can also be observed from the liquid-state ^{1}H–NMR spectra after the addition of FAI and MABr into the $[C_4mim]^+[C_8SO_4]^-$ solution, suggesting the reduced electron cloud density of protons and thus the formation of a hydrogen bond between the H atom of the amidine in FA and the N3 atom of the imidazole ring in $[C_4mim]^+$ (ref. [14]).

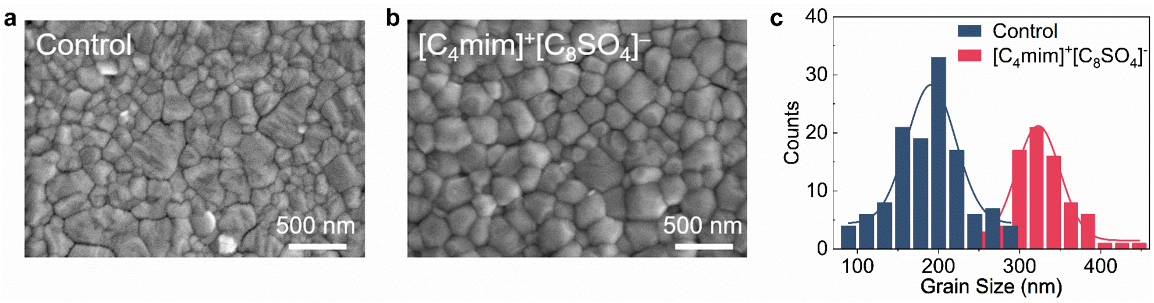

Supplementary Figure 19. SEM measurements of control and $[C_4mim]^+[C_8SO_4]^-$ treated perovskite films. (**a**,**b**) SEM top-view images of (**a**) control and (**b**) $[C_4mim]^+[C_8SO_4]^-$ treated perovskite films (scale bar, 500 nm). (**c**) Particle size distribution of corresponding perovskite films.

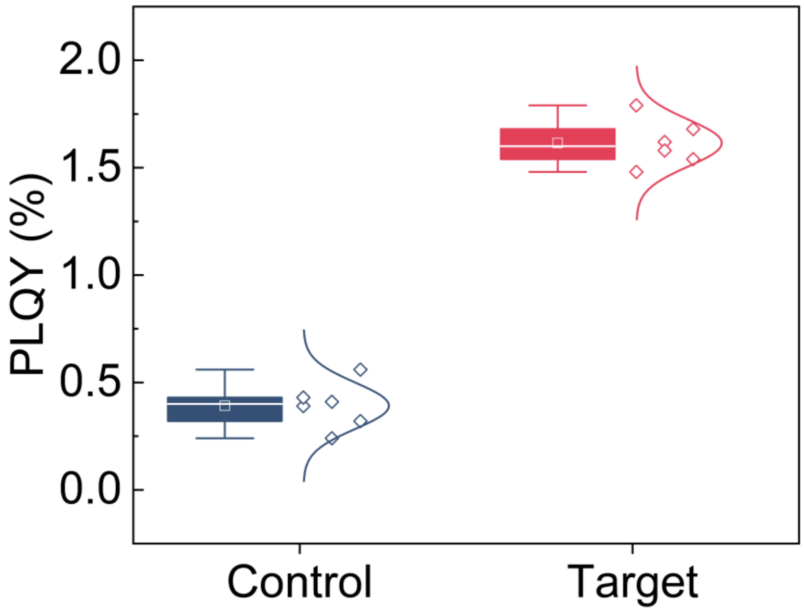

Supplementary Figure 20. PLQY values from six individual control films and target films on glass substrates. The PLQY was obtained through 532 nm photoexcitation at 24 mW/cm^2 intensity to achieve a 0.5-sun equivalent photon flux[15]. To confirm the decreased defect-mediated nonradiative recombination in the target films, we performed photoluminescence quantum yield (PLQY) measurements[16]. The PLQY increased by ~4 times to 0.39% for the target films compared with 1.61% for the control films. The results quantified the defect-mediated nonradiative recombination of photogenerated charge carriers in perovskite films and hence predict the ultimate device performance.

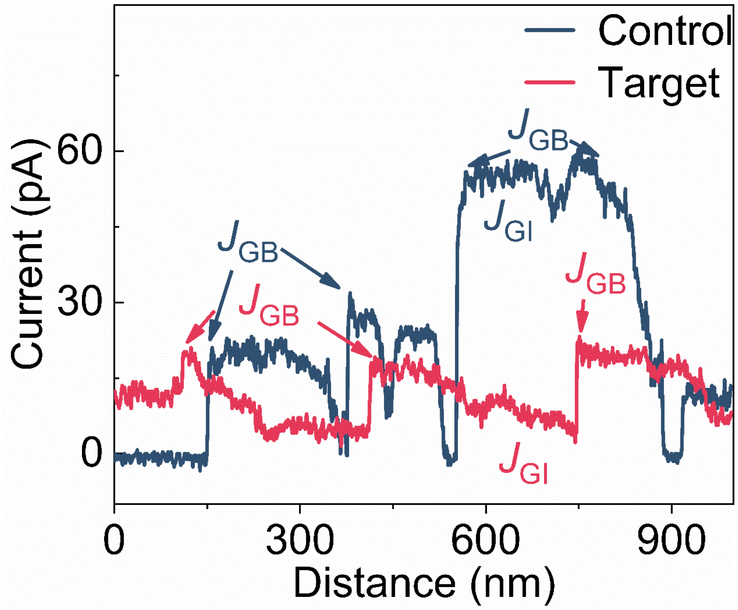

Supplementary Figure 21. C-AFM measurements. Line profiles of local current flow in the c-AFM images of control and target films.

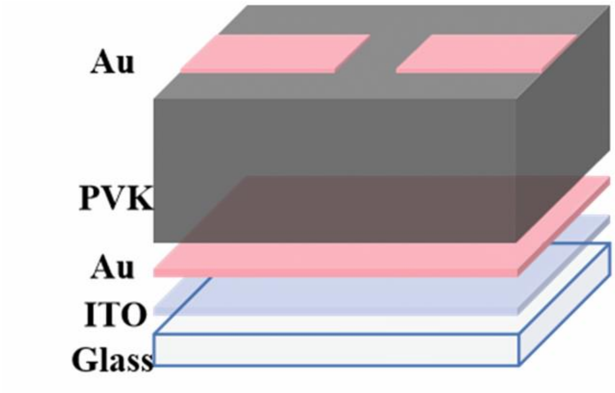

Supplementary Figure 22. Device structure for electrical poling experiments. The structure of ITO/Au/perovskite/Au for the electrical poling experiments.

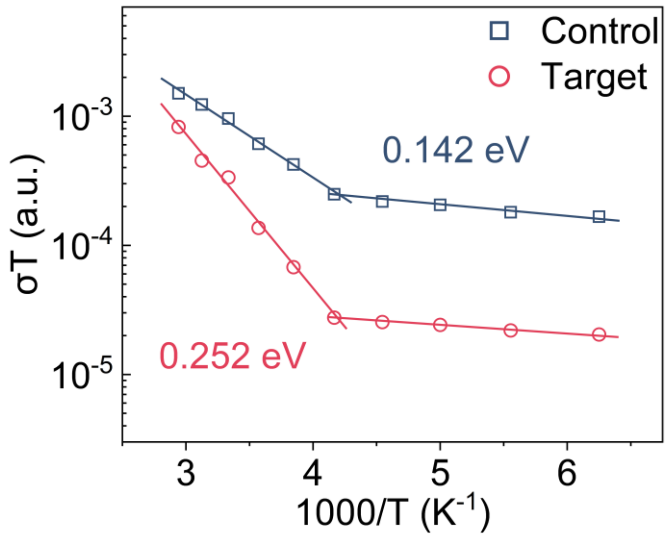

Supplementary Figure 23. Temperature-dependent conductivity of control and target perovskite films. Activation energy (*E*a) measurement of ions migration: The current was extracted at 60 s after the voltage was switched on. The measurement was conducted in a probe station (Cindbest CGO-4) under vacuum (1.0×10^{-4} Pa). The samples were placed on a substrate with temperature control by a heater and injected liquid N_2. A semiconductor characterization system (Keysight B1500A) was used for the current measurement. During the measurement, we first cooled the devices to 100 K for 1 hour and then heated to objective temperature. Every objective temperature was stabilized for 5 min before the current record. The activation energy can be extracted from the Nernst-Einstein relation: $\sigma(\mathrm{T}) = (\sigma_0/T)\exp(-Ea/k_B T)$, where k_B is the Boltzmann constant, σ_0 is a constant, and T is temperature.

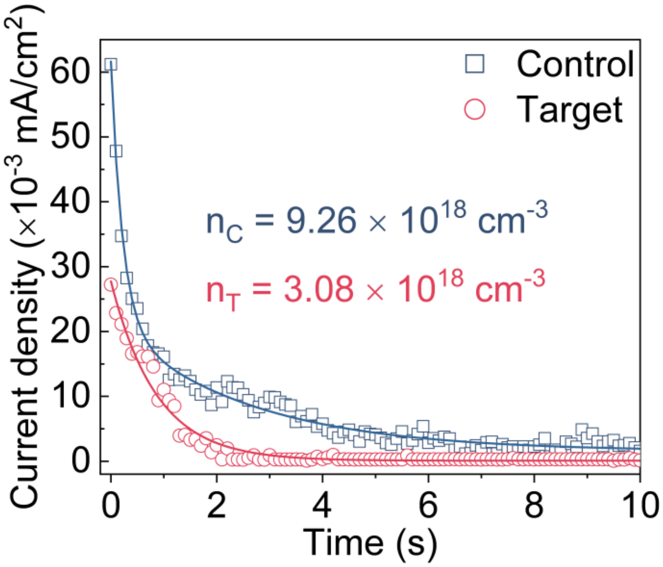

Supplementary Figure 24. Transient ionic current (TIC) for extracting mobile ion concentration within the perovskite films of the control and target devices. The reduced mobile ion concentrations can be observed in the target samples (3.08×10^{18} cm^{-3}) compared with control samples (9.26×10^{18} cm^{-3}). The concentrations of mobile ions in the perovskite films were estimated from the transient ionic current based on the following formula[17]:

$$n = \frac{\int_{t1}^{t2} J\, dt}{eL} \quad (4)$$

Where *t* is the time, *J* is the current density, *e* is the elementary charge and *L* is the thickness of the perovskite film. The transient ionic relaxation current was conducted at dark with an external forward bias at V_{OC} for 60 s. The measured current is mainly given by the redistribution of mobile ions in the perovskite layer after removing the external applied voltage because of the dark test condition.

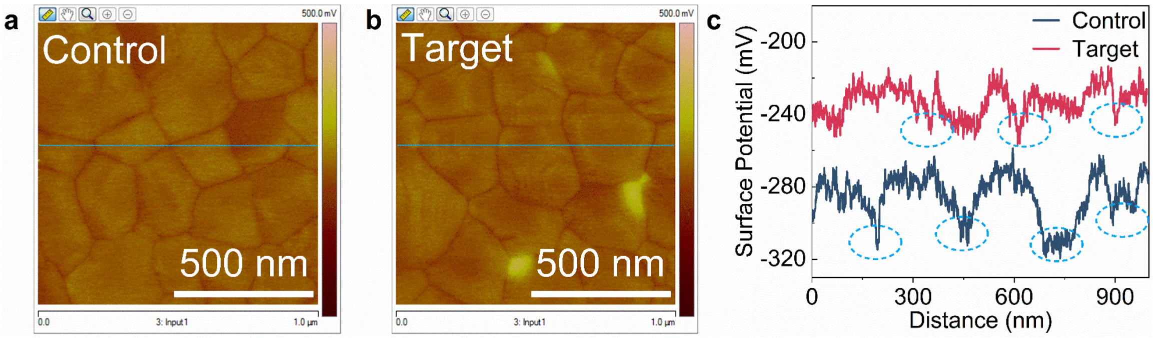

Supplementary Figure 25. Top view KPFM images of control films and target films. The contact potential differences (CPDs) of (**a**) control films and (**b**) target films are measured using a Pt/Cr coated Si tip as the reference. A positive (negative) CPD value indicates a lower (higher) work function (WF) compared to that of the biased tip. (**c**) Line profiles of local surface potential in the KPFM images of control and target films. The local GBs are indicated with the blue dotted circles. The control perovskite exhibits a relatively uniform contact potential difference (CPD) map, but there is still a CPD difference of ≈ 62 mV between the GBs and adjacent GIs, in which the local GBs show a relatively low potential (indicated by the blue dotted circles). As for the target perovskite, the CPD along the GBs significantly increased to be comparable to that of the adjacent grain interior, rendering a small CPD difference of only ≈43 mV. Notably, the overall CPD of the target perovskite (–240 mV) is much higher than that of the control one (–300 mV), confirming a more n-type perovskite surface.

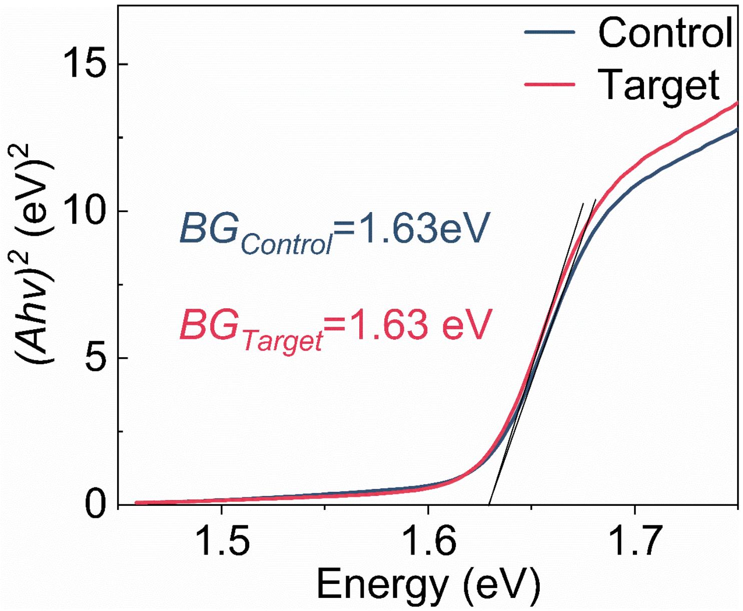

Supplementary Figure 26. Tauc plot from UV-vis absorption spectra of control and target perovskite films. Both the control and target films show the optical bandgap of 1.63 eV.

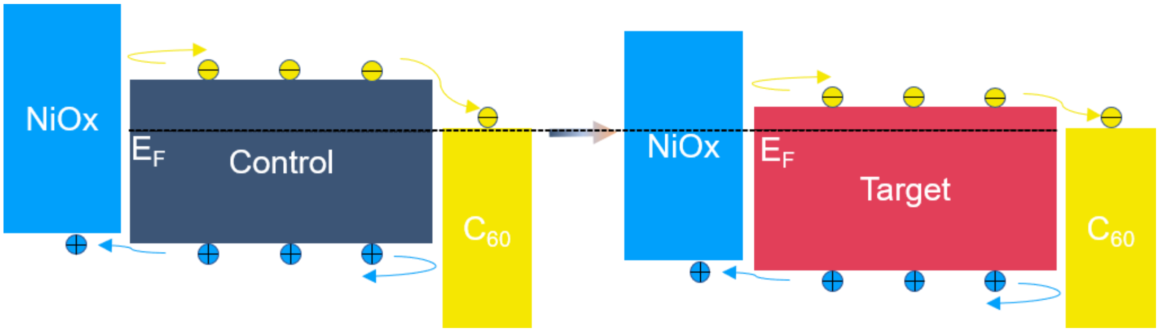

Supplementary Figure 27. Schematics of energy level. Energy level alignment schematics of the control and target PSCs.

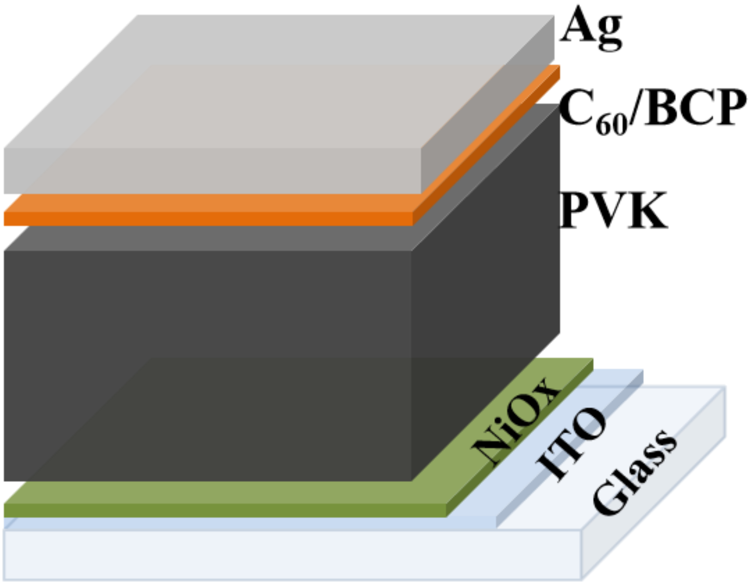

Supplementary Figure 28. Schematic of perovskite device. Schematic of the single-junction perovskite device.

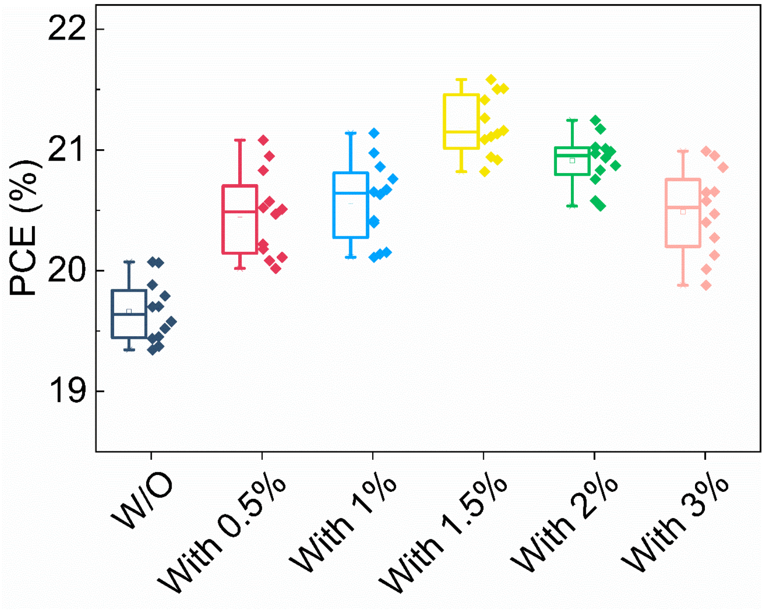

Supplementary Figure 29. Concentration optimization of $[C_4mim]^+[C_8SO_4]^-$. The box plot denotes the median (center line), 75th (top edge of the box), 25th (bottom edge of the box) percentiles. The diamond-shaped points represent the specific distribution of device efficiencies.

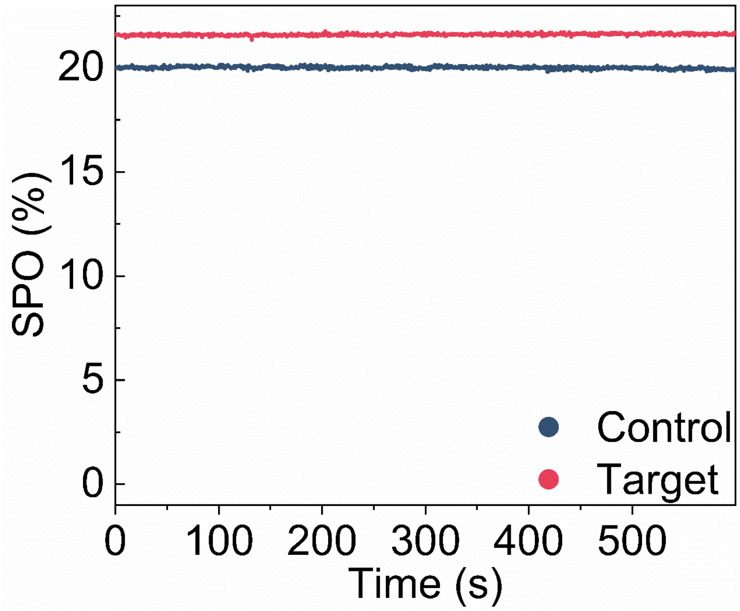

Supplementary Figure 30. The SPO of the control and target devices for 10 minutes. The V_{max} and J_{max} are 0.94 V and 21.1 mA·cm^{-2} for the control device and 0.98 V and 21.9 mA·cm^{-2} for the target device, respectively.

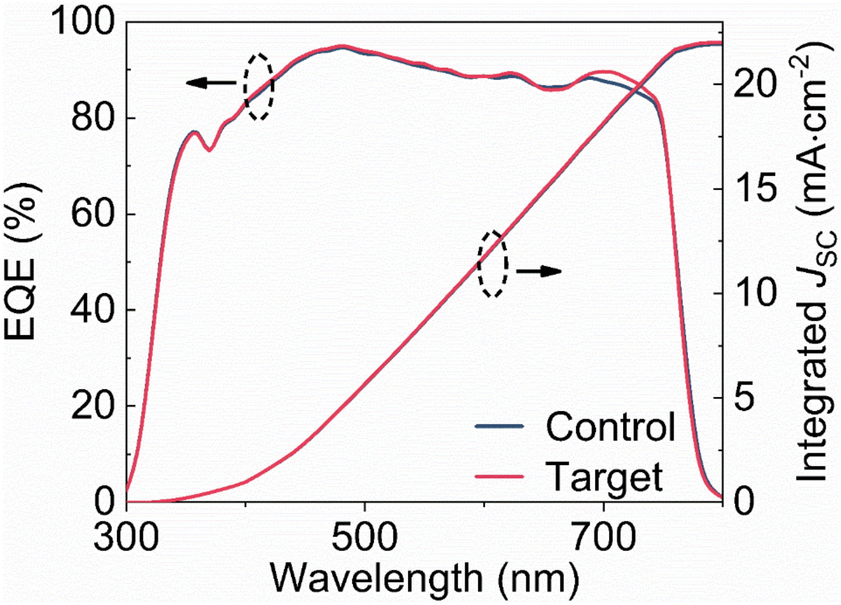

Supplementary Figure 31. EQE measurements. EQE and integrated J_{SC} of the control and target devices.

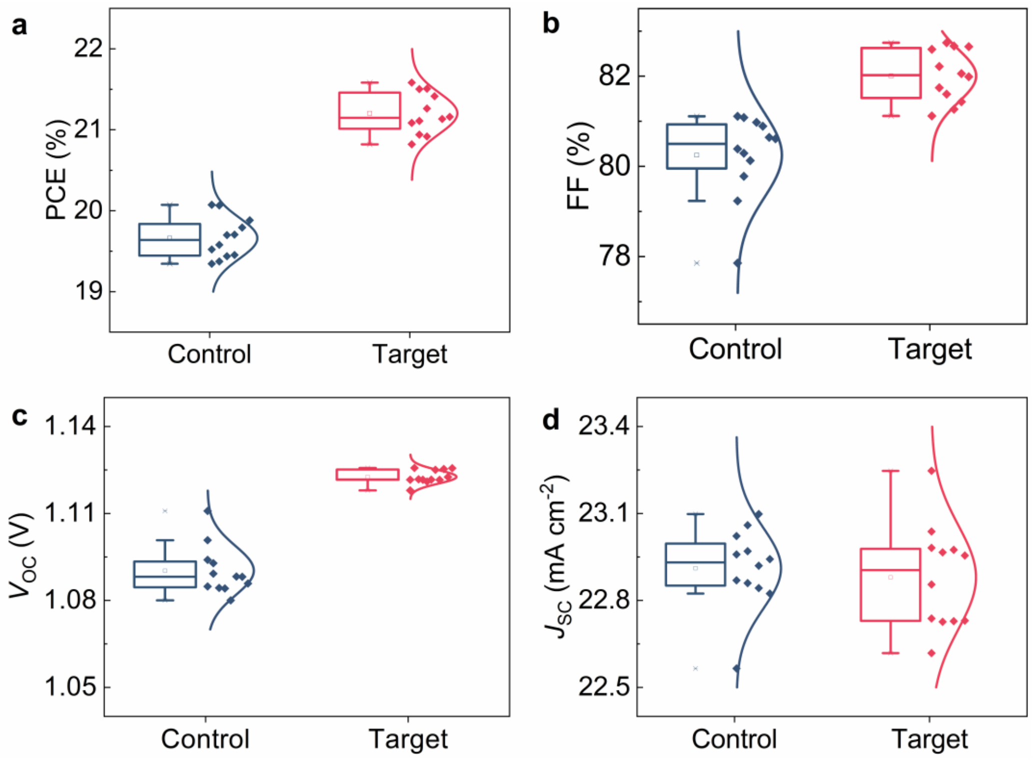

Supplementary Figure 32. The statistics of photoelectric performance parameters distribution for the 12 control and target perovskite solar cells. Performance parameters distribution for the 12 control and target devices: (**a**) PCE; (**b**) FF; (**c**) V_{OC}; (**d**) J_{SC}. The box plot denotes the median (center line), 75th (top edge of the box) and 25th (bottom edge of the box) percentiles. The colored diamond and curves are the statistical data points and corresponding normal distribution curves. All these performance parameters are obtained on the reverse scan.

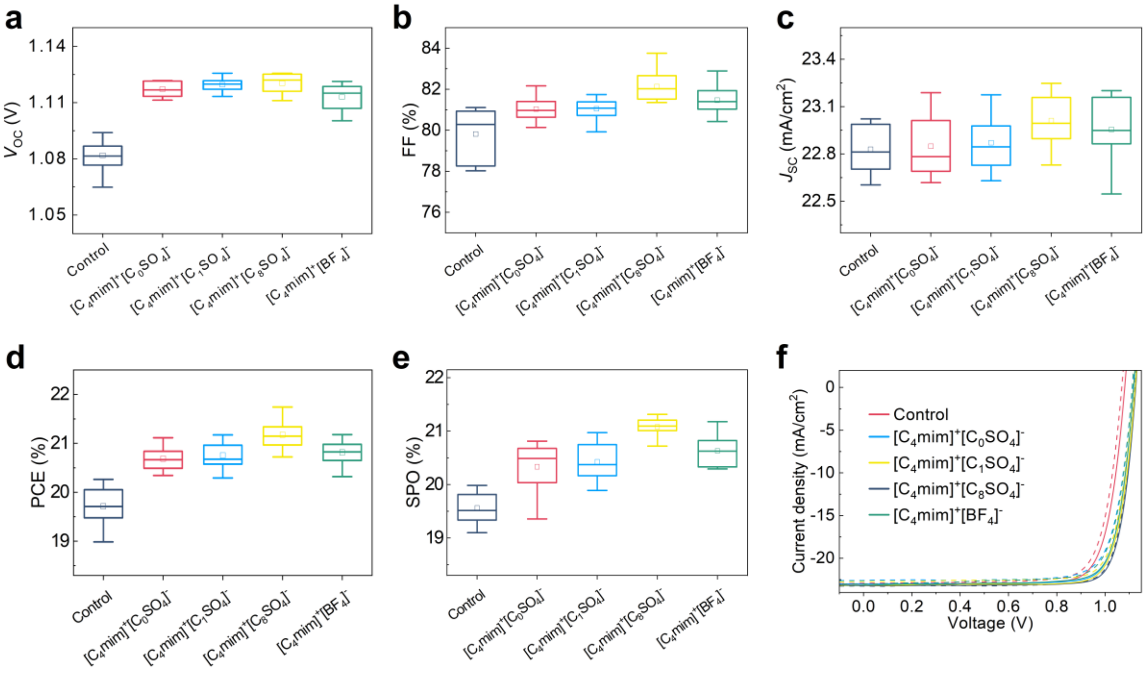

Supplementary Figure 33. The statistics of photoelectric performance parameters distribution for the perovskite solar cells. Performance parameters distribution for the control, $[C_4mim]^+[C_0SO_4]^-$, $[C_4mim]^+[C_1SO_4]^-$ and $[C_4mim]^+[C_8SO_4]^-$ and $[C_4mim]^+[BF_4]^-$ treated devices: (**a**) V_{OC}; (**b**) FF; (**c**) J_{SC}; (**d**) PCE; (**e**) SPO and (**f**) the corresponding J–V curves. The box plot denotes the median (center line), 75th (top edge of the box) and 25th (bottom edge of the box) percentiles. The colored diamond and curves are the statistical data points and corresponding normal distribution curves. All these performance parameters are obtained on the reverse scan from 12 individual devices. Compared with control devices, the device statistics corroborated that the PCE improvements were mainly the result of enhanced V_{OC} and FF in the treated devices. The improvement of $[C_4mim]^+[C_8SO_4]^-$-treated devices is the most significant, while the $[C_4mim]^+[C_0SO_4]^-$ and $[C_4mim]^+[BF_4]^-$-treated devices are accompanied with an obvious hysteresis. The differences in device performance are likely attributed to the different distribution of additives in perovskite films[18].

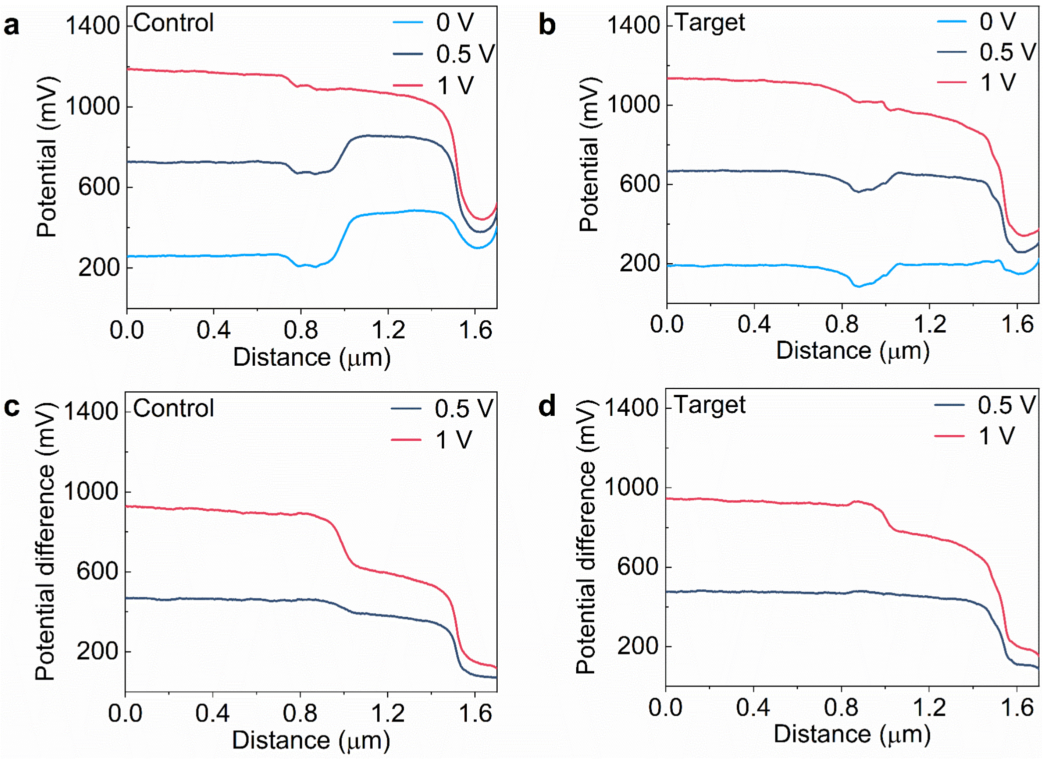

Supplementary Figure 34. Cross-section potential profiles across the whole devices. Cross-section potential profiling under different bias voltages across the whole (**a**) control device and (**b**) target device. Cross-section potential differences profiling across the (**c**) control device and (**d**) target device under different bias voltages subtracted from the 0 V curve.

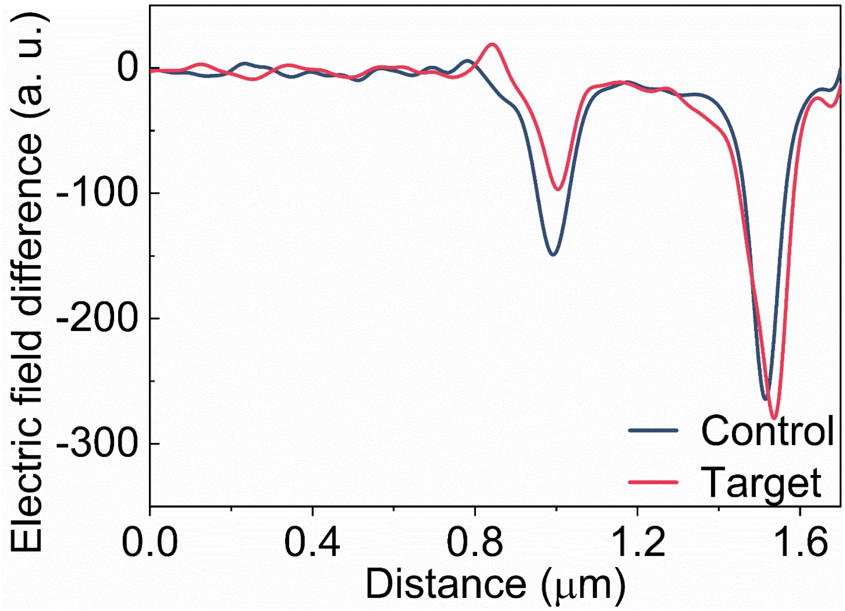

Supplementary Figure 35. The cross-sectional KPFM measurements. Electric-field differences of the control device and the target device under 1 V bias voltages.

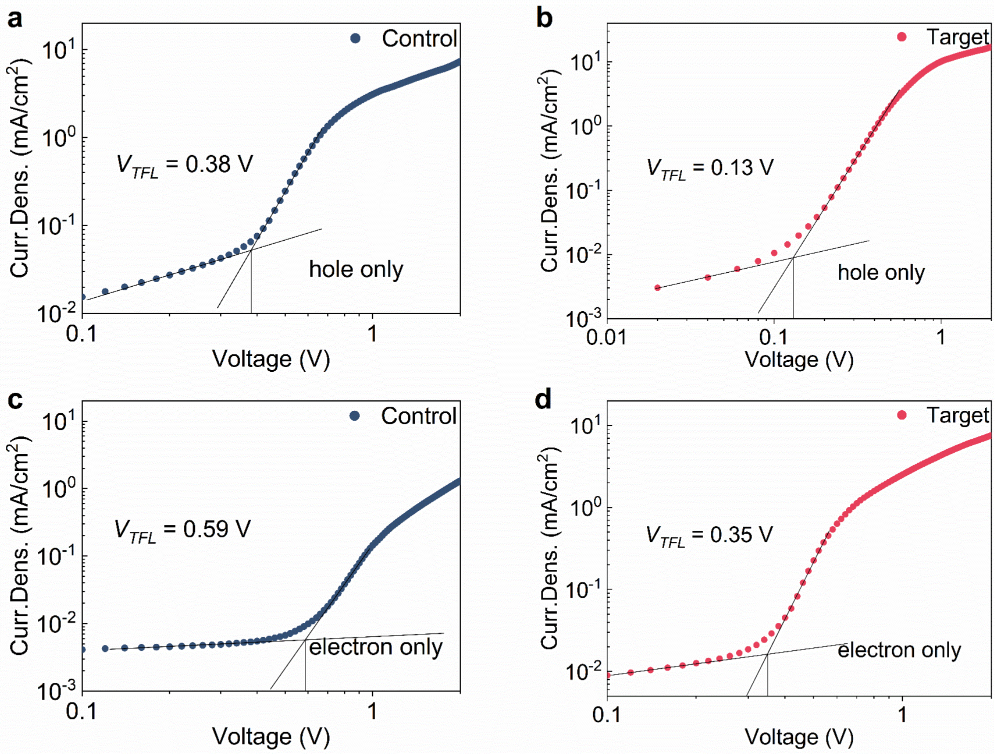

Supplementary Figure 36. SCLC measurements for control and target devices. (**a**,**b**) Hole only devices, and (**c**,**d**) electron only devices. SCLC measurements are used to quantify the defect density of control and target devices. A Keithly 2400 source meter is used to measure the relevant J–V curves. We calculate the trap density (N_t) by SCLC measurement in the hole-only and electron-only devices. The trap-state density N_t can be calculated using the following relation:

$$V_{TFL} = N_t \frac{eL^2}{2\varepsilon\varepsilon_0} \quad (5)$$

where ε_0 and ε are the vacuum permittivity and the relative dielectric constant of perovskite, respectively, N_t is the defect density, e is the electron charge, L is the thickness of the film, and V_{TFL} is the kink point in the dark J–V curve.

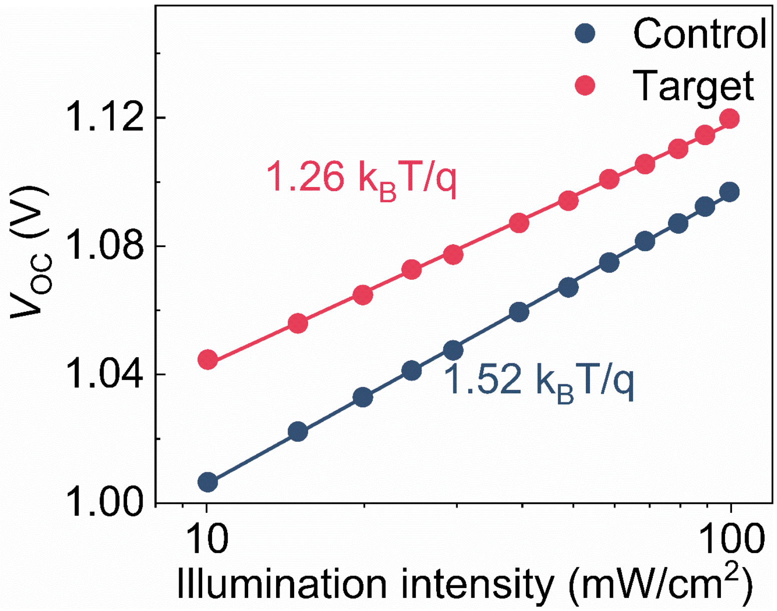

Supplementary Figure 37. Light intensity-dependent V_{OC} measurements of control and target device. We carry out light intensity-dependent V_{OC} measurements to further evaluate the defect density of control and target devices. The ideality factor will be close to 1 when radiative recombination prevails. As clear evidence for the reduced trap-assisted recombination, we examine the diode ideality factor (n) according to the following equation:

$$V_{OC} = \frac{nk_BT}{q} ln(I) + C \quad (6)$$

where k_B is the Boltzmann constant, n is the ideality factor, T is the temperature, I is light intensity and C is a constant.

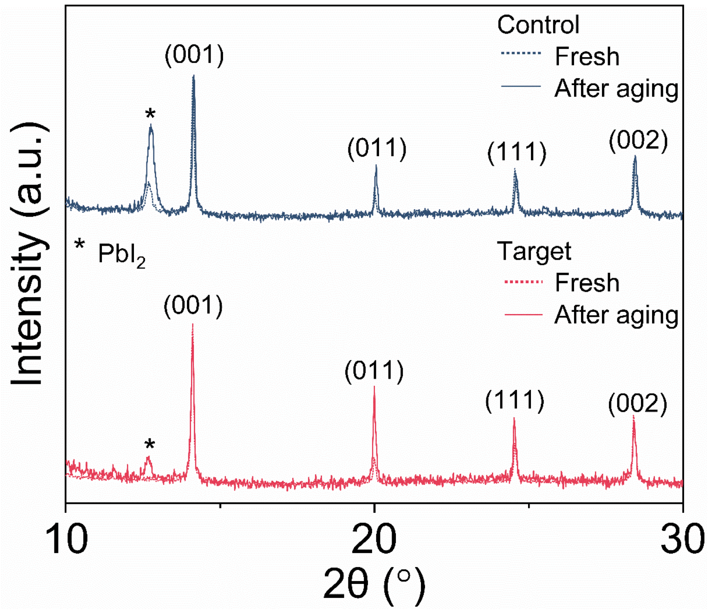

Supplementary Figure 38. Out-of-plane XRD of the control and target films before and after accelerated aging for 14 days of continuous illumination in N_2 atmosphere.

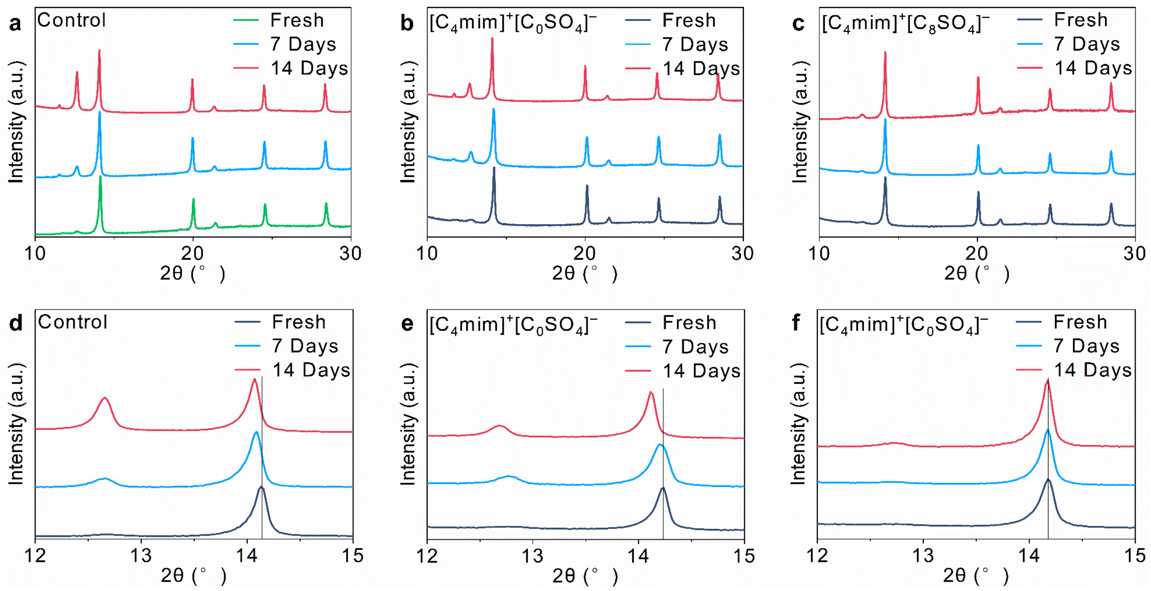

Supplementary Figure 39. Time-dependent XRD measurements of perovskite films. Time-dependent XRD measurements of (**a**) control film, (**b**) $[C_4mim]^+[C_0SO_4]^-$ treated film and (**c**) target film, and magnified diffraction peaks of (**d**) control film, (**e**) $[C_4mim]^+[C_0SO_4]^-$ treated film and (**f**) $[C_4mim]^+[C_8SO_4]^-$ treated film.

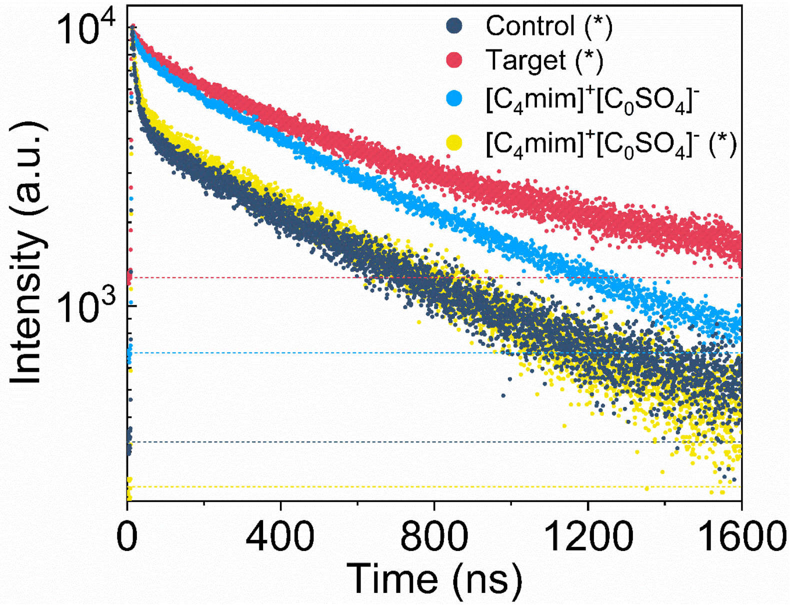

Supplementary Figure 40. TRPL decay curves for perovskite films. TRPL decay curves for control film, $[C_4mim]^+[C_0SO_4]^-$ treated film and target films (TRPL measurements after illumination are denoted with*). The dashed lines indicate the background levels.

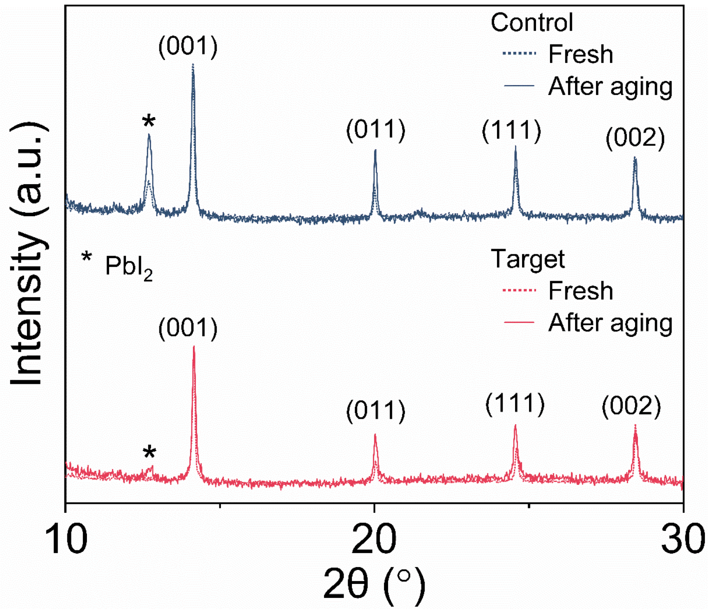

Supplementary Figure 41. XRD measurements. Out-of-plane XRD of the control and target films before and after accelerated aging of 150 °C in air for 20 minutes.

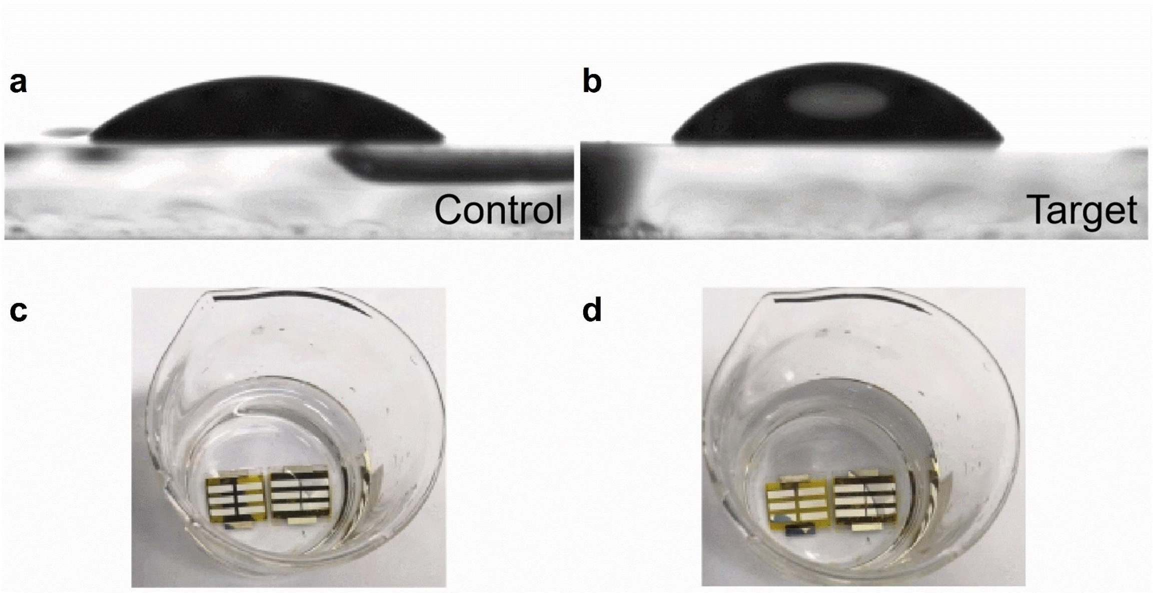

Supplementary Figure 42. The moisture stability of the perovskite. The static water contact angle (CA) of (**a**) control film and (**b**) target film. The pictures of the control device (left) and target device (right) immersed in water for (**c**) 5 seconds and (**d**) after 3 minutes.

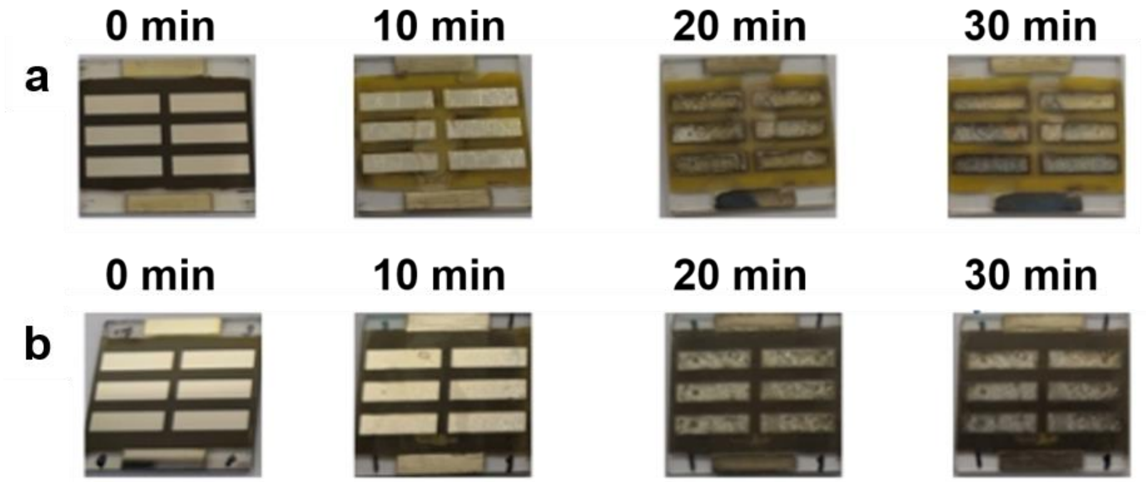

Supplementary Figure 43. Stability of the perovskite devices. The pictures of (**a**) control device and (**b**) target device stored in T = 85 °C, RH = 85% and light intensity = 100 mW·cm^{-2} for 30 minutes. The perovskite layer degrades into yellow PbI_2 in just 10 minutes for the control device, while no obvious yellow PbI_2 can be observed for the target device even after 30 minutes of aging.

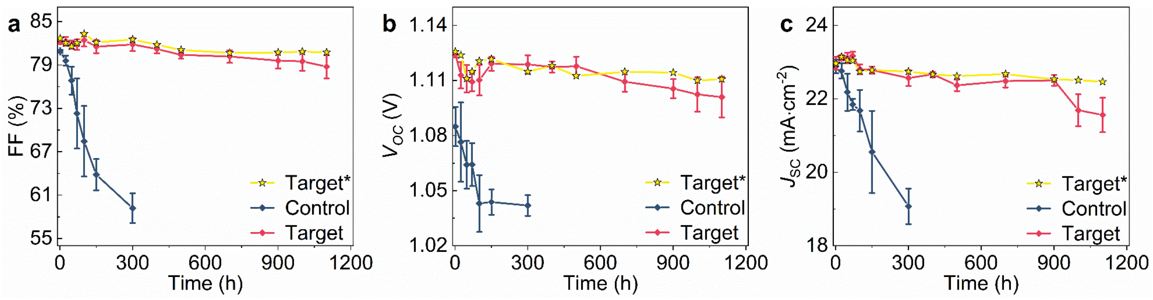

Supplementary Figure 44. Performance statistics for ISOS-L2 measurements of unencapsulated single-junction devices held at 65 °C with ~50±5% relative humidity in air. Evolution of device parameters for unencapsulated perovskite solar cells under ISOS-L2 conditions: (**a**) FF; (**b**) V_{OC}; (**c**) J_{SC}. The target champion cell (denoted with*) is indicated with yellow stars (surrounded by black border lines). Standard deviation (error bar) is calculated from six individual tandem devices in the same batch.

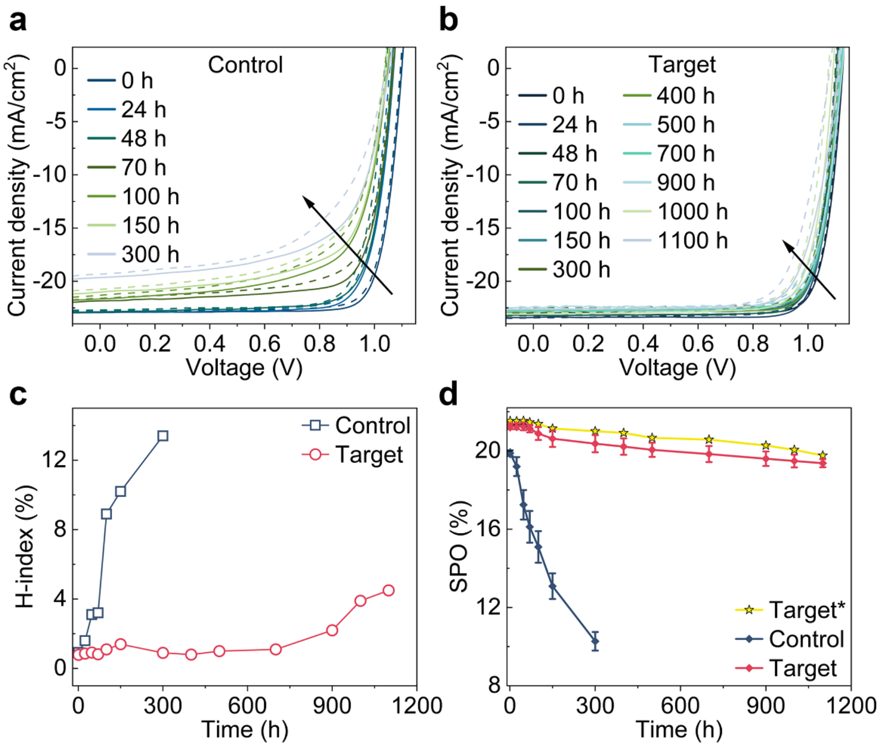

Supplementary Figure 45. Performance of control and target devices. J–V curves of (a) control and (b) target devices at different intervals during the ISOS-L2 measurements, in forward scan (dotted lines) and reverse scan (solid lines). (c) The hysteresis index for the corresponding devices during the ISOS-L2 measurements. (d) SPO of the non-encapsulated control and target devices, during aging during ISOS- L2 measurements. The target champion cell (denoted with*) is indicated with yellow stars (surrounded by black border lines). Standard deviation (error bar) is calculated from six individual devices in the same batch.

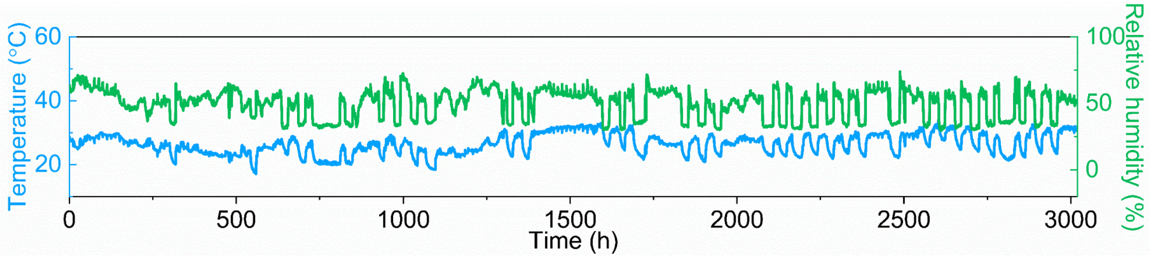

Supplementary Figure 46. Temperature and the relative humidity measurements. The ambient temperature and the relative humidity during stability testing of the unencapsulated target single-junction device.

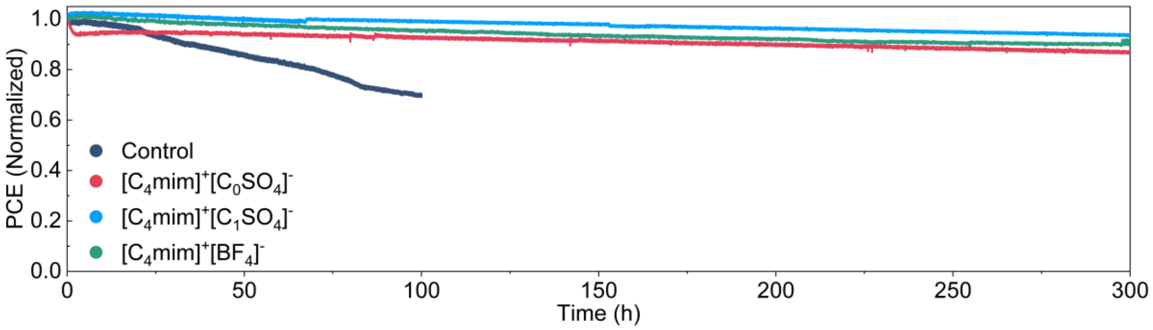

Supplementary Figure 47. The operational stability of single-junction devices. The operational stability of the unencapsulated control, $[C_4mim]^+[C_0SO_4]^-$, $[C_4mim]^+[C_1SO_4]^-$ and $[C_4mim]^+[BF_4]^-$ treated single-junction devices under MPPT in air. For the control device, the PCE quickly decreased to around 70% after roughly 100 hours of aging. By comparison, for the $[C_4mim]^+[C_0SO_4]^-$, $[C_4mim]^+[C_1SO_4]^-$ and $[C_4mim]^+[BF_4]^-$-treated cells, we observed the roughly 16%, 7% and 10% drop in the PCEs for 300 hours aging. Moreover, the early-time 'burn-in' was observed in the $[C_4mim]^+[C_0SO_4]^-$-treated cell during the MPPT measurement.

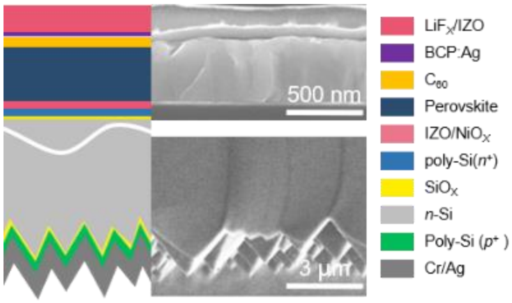

Supplementary Figure 48. The architecture and SEM of tandem device. The architecture and corresponding cross-sectional SEM image of perovskite/silicon tandem device.

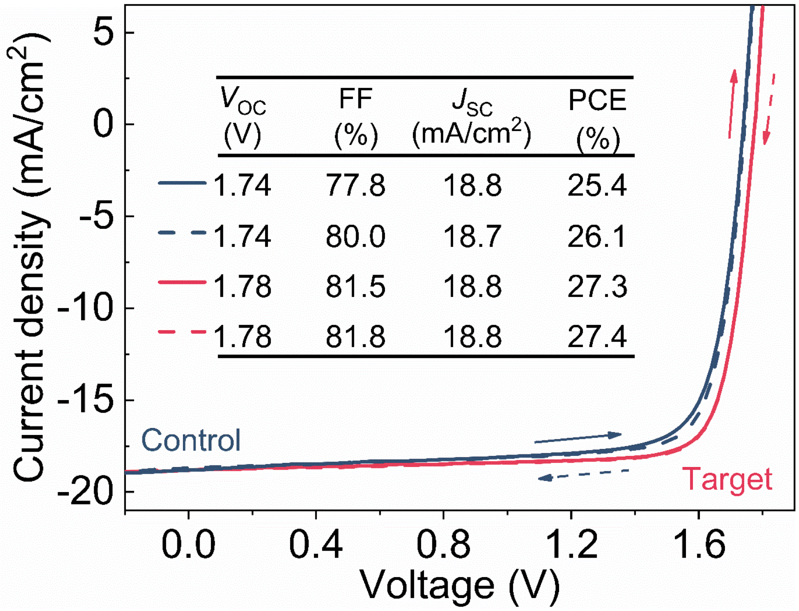

Supplementary Figure 49. J–V curves of the control and target tandem solar cells. J–V curves of the best-performing control (navy) and target (red) devices, in forward scan (solid lines) and reverse scan (dotted lines). The inset table shows the corresponding efficiency parameters.

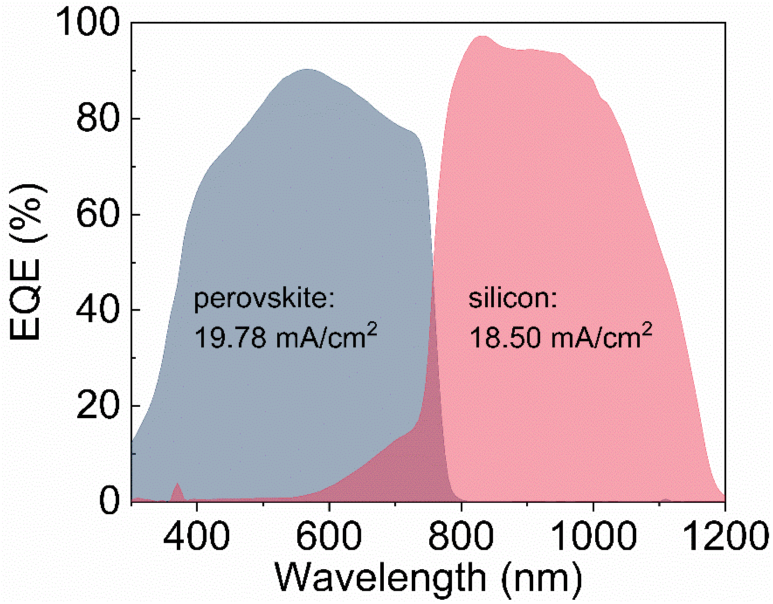

Supplementary Figure 50. EQE measurements of tandem device. EQE of perovskite/silicon tandem device and the integrated short-circuit current density of the top perovskite solar cell and bottom silicon solar cell.

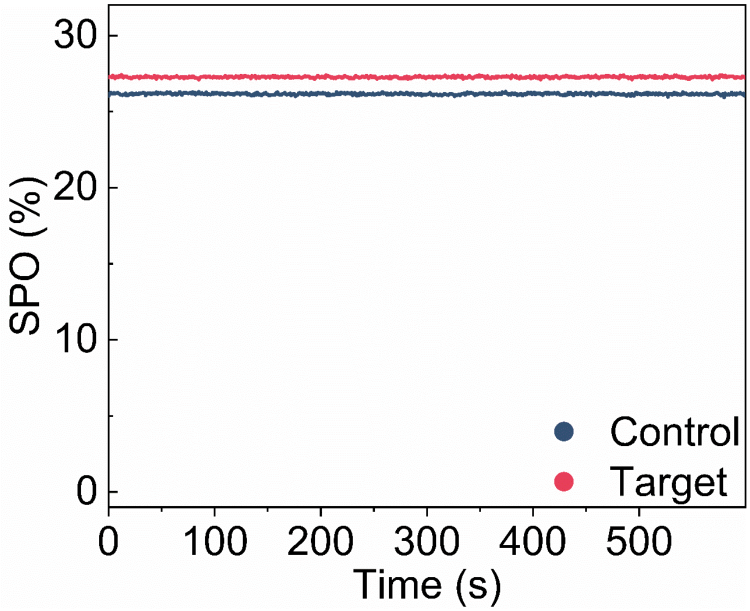

Supplementary Figure 51. The SPO of the control and target 2–T perovskite/silicon tandem devices. The V_{max} and J_{max} are 1.52 V and 17.1 mA·cm^{-2} for the control device and 1.56 V and 17.5 mA·cm^{-2} for the target device, respectively.

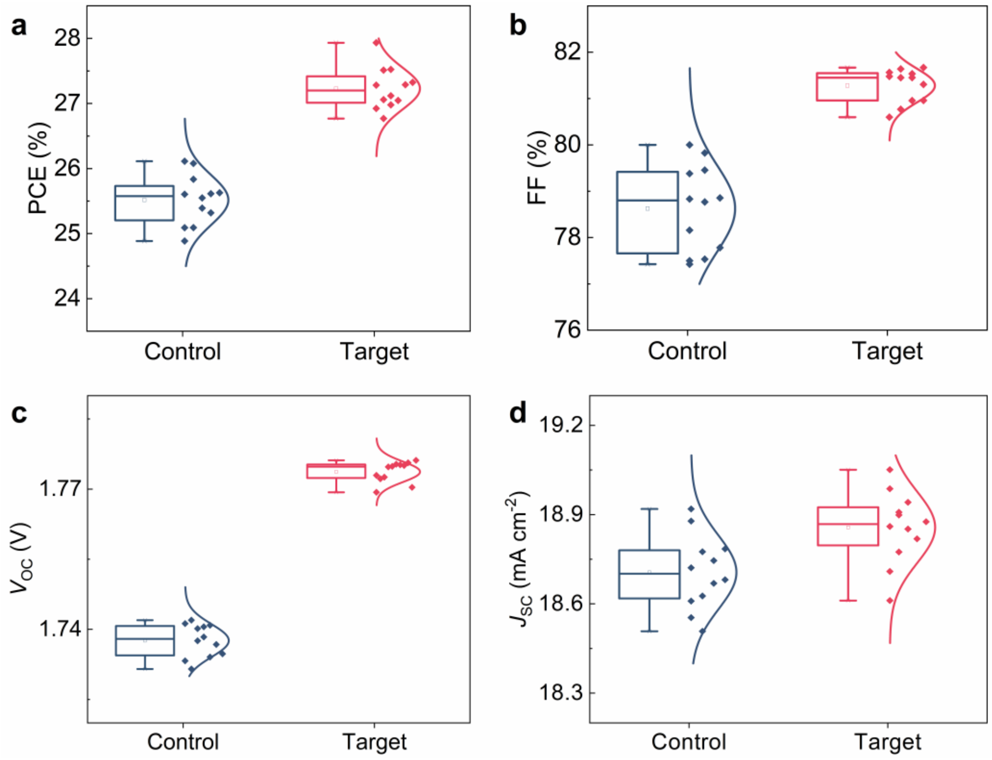

Supplementary Figure 52. The statistics of photoelectric performance parameters distribution for the 12 control and target perovskite/silicon tandem solar cells with the architecture Ag/Cr/poly-Si(p+)/SiO$_x$/n-Si/SiO$_x$/poly-Si(n+)/IZO/NiO$_x$/perovskite/C$_{60}$/BCP/IZO/LiF$_x$/Ag. Performance parameters distribution for the 12 control and target tandem devices: (**a**) PCE; (**b**) FF; (**c**) V_{OC}; (**d**) J_{SC}. The box plot denotes the median (center line), 75th (top edge of the box) and 25th (bottom edge of the box) percentiles. The colored diamond and curves are the statistical data points and corresponding normal distribution curves. All these performance parameters are obtained on the reverse scan.

中国认可
国际互认
检测
TESTING
CNAS L8490
170017163974
Test and Calibration Center of New Energy Device and Module,
Shanghai Institute of Microsystem and Information Technology,
Chinese Academy of Sciences (SIMIT)
235, Chengbei Road, Jiading, Shanghai, China

Measurement Report

**Client Name**	Ningbo Institute of Materials Technology and Engineering(NIMTE), Chinese Academy of Sciences (CAS)
**Client Address**	1219 Zhongguan West Road, Ningbo, China (315201)
**Sample**	Perovskite/Silicon Tandem Solar Cell
**Manufacturer**	Ningbo Institute of Materials Technology and Engineering(NIMTE), Jichun Ye Group
**Application**	SIMITL72022022502
**Measurement Date**	25th February, 2022

**Performed by:**	Qiang Shi<br>Qiang Shi	**Date:**	25/02/2022
**Reviewed by:**	Yating Zhang<br>Yating Zhang	**Date:**	25/02/2022
**Approved by:**	Zhengxin Liu<br>Zhengxin Liu	**Date:**	Feb. 26, 2022

检测专用章

The measurement report without signature and seal are not valid. This report shall not be reproduced, except in full, without the approval of SIMIT.

Test and Calibration Center of New Energy Device and Module

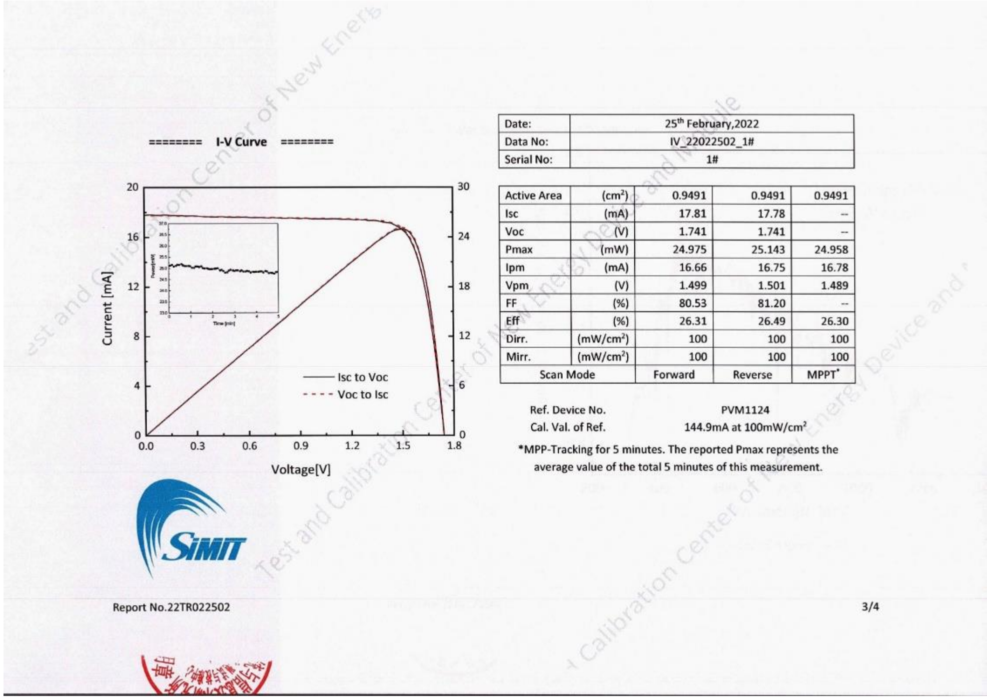

======== I-V Curve ========

Date:	25th February,2022
Data No:	IV_22022502_1#
Serial No:	1#

Active Area	(cm^2)	0.9491	0.9491	0.9491
Isc	(mA)	17.81	17.78	--
Voc	(V)	1.741	1.741	--
Pmax	(mW)	24.975	25.143	24.958
Ipm	(mA)	16.66	16.75	16.78
Vpm	(V)	1.499	1.501	1.489
FF	(%)	80.53	81.20	--
Eff	(%)	26.31	26.49	26.30
Dirr.	(mW/cm^2)	100	100	100
Mirr.	(mW/cm^2)	100	100	100
Scan Mode		Forward	Reverse	MPPT*

Ref. Device No. PVM1124

Cal. Val. of Ref. 144.9mA at 100mW/cm²

*MPP-Tracking for 5 minutes. The reported Pmax represents the average value of the total 5 minutes of this measurement.

Report No.22TR022502 3/4

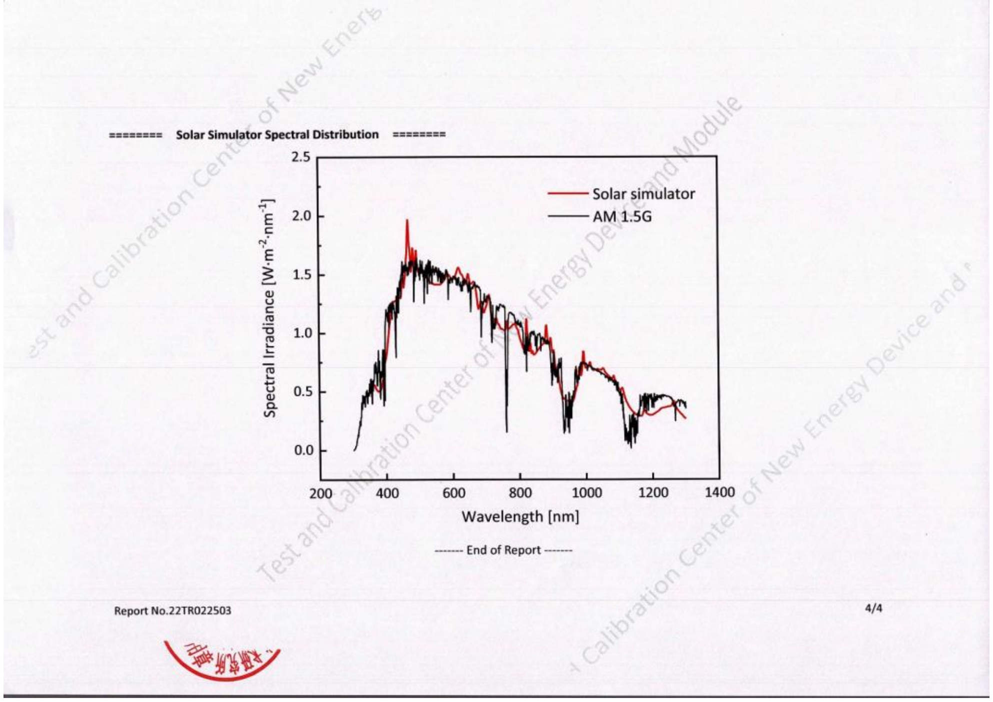

======== Solar Simulator Spectral Distribution ========

------- End of Report -------

Report No.22TR022503 4/4

Supplementary Figure 53. PV certification using dual-source solar simulator measured at SIMIT. The certified results of the monolithic perovskite/silicon tandem solar cell (unencapsulated, ~1cm^2). The device exhibits certified PCEs of 26.30% (V_{OC} = 1.741 V, J_{SC} = 18.76 mA/cm^2, FF = 80.53%) under reverse scan and of 26.49% (V_{OC} = 1.741 V, J_{SC} = 18.73 mA/cm^2, FF = 81.20%) under forward scan. After the 5 minutes of steady-state MPP tracking, the stabilized PCE of 26.30% (under a V_{MPP} of 1.489 V) was recorded.

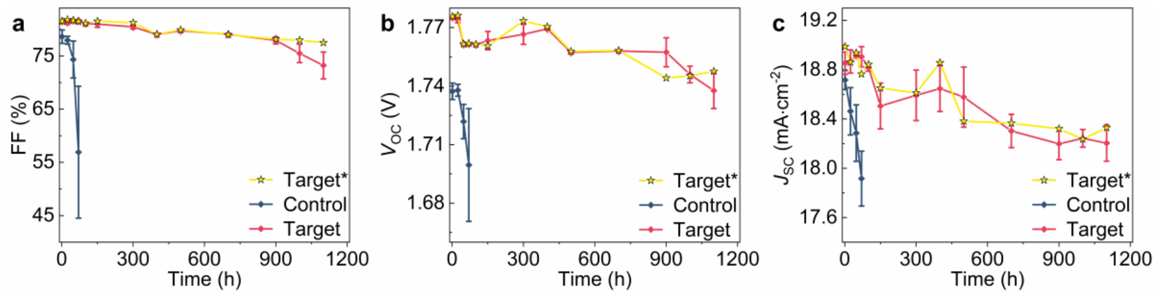

Supplementary Figure 54. Performance statistics for ISOS-L2 measurements of unencapsulated perovskite/silicon tandem devices held at 65 °C with ~50±5% relative humidity in air. Evolution of device parameters for unencapsulated tandem solar cells under ISOS-L2 conditions: (**a**) FF; (**b**) V_{OC}; (**c**) J_{SC}. The target champion tandem cell (denoted with*) is indicated with yellow stars (surrounded by black border lines). Standard deviation (error bar) is calculated from six individual tandem devices in the same batch.

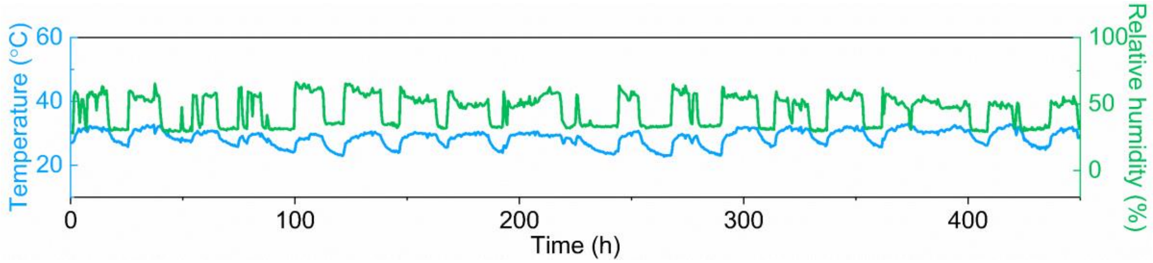

Supplementary Figure 55. Temperature and the relative humidity measurements. The ambient temperature and the relative humidity during stability testing of the unencapsulated perovskite/silicon tandem solar cells.

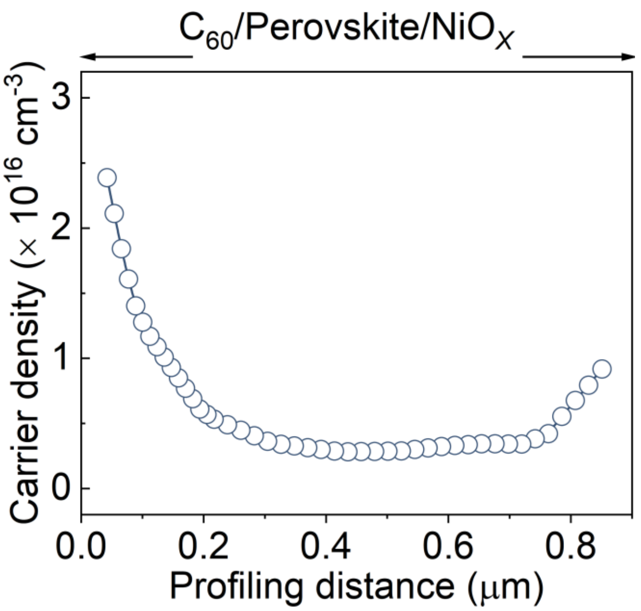

Supplementary Figure 56. The distribution of free carriers in the perovskite layer obtained by drive-level capacitance profiling (DLCP) at 1 MHz. Here, by measuring the device at 1 MHz in the dark, we observed that the carrier concentration in the region closer to the C_{60}/perovskite interface increased by up to 2 times compared with perovskite/NiO_x, indicating a more serious free carries accumulation at the poor ETL/perovskite interface[19].

Supplementary Table 1. Summary of MPP stability for perovskite solar cells reported in previous literatures.

Sample	Polarity	Environment	Encapsulation	Light condition	UV filter	PCE retention
Xu, J., et al. (Science)[20]	*p-i-n*	In ambient air; RH=25–30%; T=60 °C;	Unencapsulated	Sulfur-plasma lamp (77 mW/cm^2).	/	T_{90}=250 h
		In N_2 atmosphere; T=60 °C;				T_{96}=1,000 h
Kong, J., et al. (Nature)[21]	*n-i-p*	At room temperature;	/	AM 1.5 illumination	Without	T_{80}=500 h
Aktas, E., et al. (Energy Environ. Sci.)[22]	*p-i-n*	In N_2 atmosphere; T=25 °C;	Unencapsulated	Metal-halide lamp (100 mW/cm^2 intensity).	With	T_{95}=250 h
Zai, H., et al. (Joule)[23]	*n-i-p*	In N_2 atmosphere; At room temperature;	Unencapsulated	White LED lamp (100 mW/cm^2).	/	T_{97}=2,000 h
		In ambient air; RH=20–30%; T=20–30 °C;				T_{96}=1,000 h
Chen, S., et al. (Science)[24]	*p-i-n*	RH=~40±10 %; T=~60 °C;	Encapsulated	Plasma lamp (100 mW/cm^2).	Without	T_{100}=550 h
Cao, Q., et al. (Energy Environ. Sci.)[25]	*p-i-n*	In ambient air; T=45°C;	Encapsulated	White LED lamp (100 mW/cm^2).	Without	$T_{93.4}$=1,000 h
Zhang, C., et al. (Nat. Energy)[26]	*n-i-p*	In N_2 atmosphere; T=25 °C;	Unencapsulated	White LED lamp.	/	T_{98}=1,300 h
		In N_2 atmosphere; T=60 °C;				T_{95}=1,300 h
Zhao, Y., et al. (Nat. Energy)[27]	*n-i-p*	In N_2 atmosphere; T=60–65 °C;	Encapsulated	Metal-halide lamp (83 mW/cm^2).	With	$T_{>100}$=1,400 h
Zhang, F., et al. (Science)[28]	*n-i-p*	In N_2 atmosphere; T=~40 °C;	Unencapsulated	White LED lamp (100 mW/cm^2).	/	T_{90}=1,000 h

Li, X., et al. (Science)[29]	*p-i-n*	T=55±5 °C;	Unencapsulated	White LED lamp (100 mW/cm^2).	/	$T_{90.5}$=1,000 h
Lin, R., et al. (Nature)[30]	*p-i-n*	In ambient air; RH=30–50%; T=25 °C;	Encapsulated	White LED lamp (100 mW/cm^2).	Without	T_{97}=350 h
Azmi, R., et al. (Science)[31]	*p-i-n*	In ambient air; T=~40 °C;	Encapsulated	AM1.5 illumination	/	T_{95}=500 h
Sanchez-Diaz, J., et al. (Joule)[32]	*p-i-n*	In N_2 atmosphere;	Unencapsulated	Xenon lamp (100 mW/cm^2).	With	T_{96}=1,300 h
Chen, H., et al. (Nat. Photonics)[33]	*p-i-n*	In N_2 atmosphere; Room temperature (RT)	Unencapsulated	White LED lamp (100 mW/cm^2).	/	$T_{104.9}$=1,000 h
		RH=50±10%; T=65 °C;	Encapsulated			T_{92}=500 h
Tan, S., et al. (Nature)[34]	*n-i-p*	In ambient air; RH= 40%; T= 40 °C	Encapsulated	AM 1.5 illumination	Without	$T_{91.5}$=800 h
Li, Z., et al. (Science)[35]	*p-i-n*	In N_2 atmosphere; T=25 °C;	Unencapsulated	White LED lamp (100 mW/cm^2).	/	T_{98}=1,500 h
Lin, X., et al. (Nat. Energy)[36]	*n-i-p*	In ambient air;	Encapsulated	AM 1.5 illumination.		T_{95}=5,000 h
Zhang, T., et al. (Science)[37]	*n-i-p*	In N_2 atmosphere; T=25 °C	Unencapsulated	AM 1.5 illumination.	/	T_{95}=500 h
**This work**	***p-i-n***	**In ambient air; RH=40–70%; T=20–35 °C;**	**Unencapsulated**	**Xenon lamp (100 mW/cm^2).**	**Without**	**$T_{85.7}$=3,000h**

Supplementary Table 2. Summary of MPP stability for monolithic perovskite/silicon tandems reported in press releases or the literature.

Simple	Polarity	Environment	Encapsulation	Light condition	PCE retention
Sahli, F., et al. (Nat. Mater.)[38]	*p-i-n*	In ambient air; RH=20–30%; T=25 °C;	Unencapsulated	Two-lamp (halogen and xenon) (100 mW/cm^2).	T_{92}=61h
		In ambient air; RH=20–30%; T=30 °C;	Encapsulated	Solaronix degradation system (100 mW/cm^2).	T_{90}=270 h
Chen, B., et al. (Joule)[39]	*p-i-n*	In N_2 atmosphere;	Encapsulated	AM 1.5 illumination.	$T_{91.5}$=250 h
Chen, B., et (Joule)[40]	*p-i-n*	In ambient air; RH=25–35%; T=30 °C;	Unencapsulated	AM 1.5 illumination.	T_{92}=100 h
Hou, Y., et al. (Science)[41]	*p-i-n*	RH=40–50%; T=20°C –40 °C;	Encapsulated	Metal halide lamp (100 mW/cm^2).	T_{100}=400 h
Al-Ashouri, A., et al. (Science)[42]	*p-i-n*	In ambient air; RH=30–40%; T=25 °C;	Unencapsulated	LED with wavelengths of 470 and 940 nm.	$T_{95.5}$=300 h
Aydin, E., et al. (Energy Environ. Sci.)[43]	*n-i-p*	In ambient air; T=25 °C;	Encapsulated	Xenon lamp (100 mW/cm^2).	T_{90}=150 h
Zhumagali, S., et al. (Adv. Energy Mater.)[44]	*p-i-n*	In ambient air; T=38 °C;	Encapsulated	Metal halide lamps (100 mW/cm^2).	T_{80}=258 h
Li, Y., et al. (Adv. Energy Mater)[45]	*p-i-n*	In N_2 atmosphere; T=25(±1) °C;	Unencapsulated	AM 1.5 illumination.	T_{90}=100 h
Liu, J., et al. (Joule)[46]	*p-i-n*	In ambient air; RH=55%; T=25 °C;	Encapsulated	Xenon-lamp.	T_{100}=250 h
Liu, K., et al. (J. Mater. Chem. A)[47]	*p-i-n*	In ambient air; T=55 °C;	Unencapsulated	AM 1.5 illumination	T_{100}=2 h
Li, R., et al. (Adv. Mater.)[48]	*p-i-n*	In ambient air; T=25 °C; RH=20%;	Unencapsulated	AM 1.5 illumination.	T_{98}=300 h

Zheng, X., et al. (ACS Energy Lett.)[49]	*p-i-n*	T=25 °C;	Encapsulated	Xenon lamp (100 mW/cm^2).	T_{100}=200 h
Wu, Y., et al. (Adv. Energy Mater)[50]	*p-i-n*	T=25 °C;	/	Solar simulator system (100 mW/cm^2).	T_{100}=4.1 h
Liu, J., et al. (Science)[51]	*p-i-n*	In ambient air;	Unencapsulated	AM 1.5G illumination.	T_{99}=4.3 h
Ren, N., et al. (Appl. Phys. Lett.)[52]	*p-i-n*	In N_2 atmosphere; T=25(±)1°C	Unencapsulated	AM 1.5G illumination.	$T_{118.2}$=100 h
Yang, G., et al. (Nat. Photonics)[53]	*p-i-n*	In ambient air; T=25 °C;	Encapsulated	Xenon lamp (100 mW/cm^2).	T_{93}=550 h
Ji, S.G., et al. (Joule)[54]	*p-i-n*	In N_2 atmosphere; T=25 °C;	Unencapsulated	White LED light source (940 nm LED light for extra compensation).	T_{98}=200 h
**This work**	***p-i-n***	**In ambient air; RH=40–70%; T=20–35 °C;**	**Unencapsulated**	**Xenon lamp (100 mW/cm^2).**	**$T_{93.6}$=450 h**

Supplementary Table 3. Surface and bulk trap densities of control and target perovskite samples.

	Surface trap densities (× 10^{17}cm^{-3})	Bulk trap densities (× 10^{17}cm^{-3})
Control films	3.57	0.23
Target films	2.17	0.14

Supplementary Table 4. Summary of photovoltaic parameters for high-performance perovskite solar cells with a bandgap of 1.63 eV.

Sample	Date	V_{OC} (V)	FF (%)	J_{SC} (mA/cm^2)	PCE (%)	SPO (%)	ref
Saliba M, et al. (Science)	2016.08.29	1.18	81.0	22.8	21.8	21.6	55
Dagar J, et al. (ACS Appl. Mater. Interfaces)	2021.03.15	1.18	83.3	22.7	22.3	22.0	56
Liu X, et al. (Nano Energy)	2021.05.04	1.13	81.5	22.1	20.4	--	57
Aktas E, et al. (Energy Environ. Sci.)	2021.05.21	1.16	80.0	22.6	21.0	21.0	58
Yang G, et al. (Nat. Photonics)	2021.07.05	1.24	77.6	22.4	21.5	21.3	59
Li Y, et al. (Adv. Mater.)	2021.12.09	1.24	77.8	22.9	22.1	21.7	60
Zheng Y, et al. (Adv. Funct. Mater.)	2022.04.24	1.18	81.8	22.6	21.8	21.1	61
Jiang J, et al. (Mater. Adv.)	2022.06.13	1.20	82.4	21.7	21.5	20.5	62
Wang C, et al. (Nat. Energy)	2022.07.21	1.15	77.8	22.2	19.8	--	63
Li B, et al. (Adv. Energy Mater.)	2022.10.30	1.20	83.0	22.1	21.9	21.3	64
Almora O, et al. (Adv. Energy Mater.)	2022.11.07	1.13	76.8	23.4	20.3	20.5	65
Castriotta LA, et al. (Nano Energy)	2023.02.08	1.14	83.2	21.5	20.3	--	66
This work		1.13	82.7	23.1	21.6	21.4	

Supplemental References

1. Rolston, N., *et al.* Engineering stress in perovskite solar cells to improve stability. *Adv. Energy Mater.* **8**, 1802139 (2018).
2. Zhao, J., *et al.* Strained hybrid perovskite thin films and their impact on the intrinsic stability of perovskite solar cells. *Sci. Adv.* **3**, eaao5616 (2017).
3. Li, X., *et al.* Improved performance and stability of perovskite solar cells by crystal crosslinking with alkylphosphonic acid omega-ammonium chlorides. *Nat. Chem.* **7**, 703-711 (2015).
4. Zhu, C., *et al.* Strain engineering in perovskite solar cells and its impacts on carrier dynamics. *Nat. Commun.* **10**, 815 (2019).
5. Zhao, Y., *et al.* Strain-activated light-induced halide segregation in mixed-halide perovskite solids. *Nat. Commun.* **11**, 6328 (2020).
6. Badrooj, M., Jamali-Sheini, F. & Torabi, N. Optoelectronic properties of mixed Sn/Pb perovskite solar cells: the study of compressive strain by raman modes. *J. Phys. Chem. C* **124**, 27136-27147 (2020).
7. Yan, S. X., *et al.* Organic molecule assisted growth of perovskite films consisting of square grains by surface-confined process. *Nanomaterials* **11**, 473 (2021).
8. Jariwala, S., *et al.* Local crystal misorientation influences non-radiative recombination in halide perovskites. *Joule* **3**, 3048-3060 (2019).
9. Zhan, Y., *et al.* Elastic lattice and excess charge carrier manipulation in 1D-3D perovskite solar cells for exceptionally long-term operational stability. *Adv. Mater.* **33**, 2105170 (2021).
10. Fu, S., *et al.* Efficient passivation with lead pyridine-2-carboxylic for high-performance and stable perovskite solar cells. *Adv. Energy Mater.* **9**, 1901852 (2019).
11. Xiao, Z., *et al.* Giant switchable photovoltaic effect in organometal trihalide perovskite devices. *Nat. Mater.* **14**, 193-198 (2015).
12. Huang, H. H., *et al.* A simple one-step method with wide processing window for high-quality perovskite mini-module fabrication. *Joule* **5**, 958-974 (2021).
13. Lee, J.-W., *et al.* Tuning molecular interactions for highly reproducible and efficient formamidinium perovskite solar cells via adduct approach. *J. Am. Chem. Soc.* **140**, 6317-6324 (2018).
14. Wang, S., *et al.* Polymeric room-temperature molten salt as a multifunctional additive toward highly efficient and stable inverted planar perovskite solar cells. *Energy Environ. Sci.* **13**, 5068-5079 (2020).
15. Lohmann, K. B., *et al.* Solvent-free method for defect reduction and improved performance of p-i-n vapor-deposited perovskite solar cells. *ACS Energy Lett.* **7**, 1903-1911 (2022).
16. Hu, S., *et al.* Synergistic surface modification of tin-lead perovskite solar cells. *Adv. Mater.*, 10.1002/adma.202208320 (2023).
17. Zhao, Y., *et al.* Anomalously large interface charge in polarity-switchable photovoltaic devices: an indication of mobile ions in organic–inorganic halide perovskites. *Energy Environ. Sci.* **8**, 1256-1260 (2015).
18. Kang, Y.-J. & Na, S.-I. Multi-site passivation-based antisolvent additive engineering with

gradient distribution for superior triple cation P-I-N perovskite solar cells. *Nano Energy* **97**, 107193 (2022).
19. Deng, Y.*, et al.* Reduced self-doping of perovskites induced by short annealing for efficient solar modules. *Joule* **4**, 1949-1960 (2020).
20. Xu, J.*, et al.* Triple-halide wide-band gap perovskites with suppressed phase segregation for efficient tandems. *Science* **367**, 1097-1104 (2020).
21. Kong, J.*, et al.* CO_2 doping of organic interlayers for perovskite solar cells. *Nature* **594**, 51-56 (2021).
22. Aktas, E.*, et al.* Understanding the perovskite/self-assembled selective contact interface for ultra-stable and highly efficient p-i-n perovskite solar cells. *Energy Environ. Sci.* **14**, 3976-3985 (2021).
23. Zai, H.*, et al.* Sandwiched electrode buffer for efficient and stable perovskite solar cells with dual back surface fields. *Joule* **5**, 2148-2163 (2021).
24. Chen, S., Dai, X., Xu, S., Jiao, H., Zhao, L. & Huang, J. Stabilizing perovskite-substrate interfaces for high-performance perovskite modules. *Science* **373**, 902-907 (2021).
25. Cao, Q.*, et al.* Star-polymer multidentate-cross-linking strategy for superior operational stability of inverted perovskite solar cells at high efficiency. *Energy Environ. Sci.* **14**, 5406-5415 (2021).
26. Zhang, C.*, et al.* Ti_1–graphene single-atom material for improved energy level alignment in perovskite solar cells. *Nat. Energy* **6**, 1154-1163 (2021).
27. Zhao, Y.*, et al.* A bilayer conducting polymer structure for planar perovskite solar cells with over 1,400 hours operational stability at elevated temperatures. *Nat. Energy* **7**, 144-152 (2022).
28. Zhang, F.*, et al.* Metastable Dion-Jacobson 2D structure enables efficient and stable perovskite solar cells. *Science* **375**, 71-76 (2022).
29. Li, X., Zhang, W., Guo, X., Lu, C., Wei, J. & Fang, J. Constructing heterojunctions by surface sulfidation for efficient inverted perovskite solar cells. *Science* **375**, 434-437 (2022).
30. Lin, R.*, et al.* All-perovskite tandem solar cells with improved grain surface passivation. *Nature* **603**, 73-78 (2022).
31. Azmi, R.*, et al.* Damp heat-stable perovskite solar cells with tailored-dimensionality 2D/3D heterojunctions. *Science* **376**, 73-77 (2022).
32. Sanchez-Diaz, J.*, et al.* Tin perovskite solar cells with >1,300 h of operational stability in N_2 through a synergistic chemical engineering approach. *Joule* **6**, 861-883 (2022).
33. Chen, H.*, et al.* Quantum-size-tuned heterostructures enable efficient and stable inverted perovskite solar cells. *Nat. Photonics* **16**, 352-358 (2022).
34. Tan, S.*, et al.* Stability-limiting heterointerfaces of perovskite photovoltaics. *Nature* **605**, 268-273 (2022).
35. Li, Z.*, et al.* Organometallic-functionalized interfaces for highly efficient inverted perovskite solar cells. *Science* **376**, 416-422 (2022).
36. Lin, X.*, et al.* In situ growth of graphene on both sides of a Cu–Ni alloy electrode for perovskite solar cells with improved stability. *Nat. Energy* **7**, 520-527 (2022).

37. Zhang, T., *et al.* Ion-modulated radical doping of spiro-OMeTAD for more efficient and stable perovskite solar cells. *Science* **377**, 495-501 (2022).
38. Sahli, F., *et al.* Fully textured monolithic perovskite/silicon tandem solar cells with 25.2% power conversion efficiency. *Nat Mater* **17**, 820-826 (2018).
39. Chen, B., *et al.* Grain engineering for perovskite/silicon monolithic tandem solar cells with efficiency of 25.4%. *Joule* **3**, 177-190 (2019).
40. Chen, B., *et al.* Blade-coated perovskites on textured silicon for 26%-efficient monolithic perovskite/silicon tandem solar cells. *Joule* **4**, 850-864 (2020).
41. Hou, Y., *et al.* Efficient tandem solar cells with solution-processed perovskite on textured crystalline silicon. *Science* **367**, 1135-1140 (2020).
42. Al-Ashouri, A., *et al.* Monolithic perovskite/silicon tandem solar cell with >29% efficiency by enhanced hole extraction. *Science* **370**, 1300-1309 (2020).
43. Aydin, E., *et al.* Ligand-bridged charge extraction and enhanced quantum efficiency enable efficient n-i-p perovskite/silicon tandem solar cells. *Energy Environ. Sci.* **14**, 4377-4390 (2021).
44. Zhumagali, S., *et al.* Linked nickel oxide/perovskite interface passivation for high-performance textured monolithic tandem solar cells. *Adv. Energy Mater.* **11**, 2101662 (2021).
45. Li, Y., *et al.* Wide bandgap interface layer induced stabilized perovskite/silicon tandem solar cells with stability over ten thousand hours. *Adv. Energy Mater.* **11**, 202102046 (2021).
46. Liu, J., *et al.* 28.2%-efficient, outdoor-stable perovskite/silicon tandem solar cell. *Joule* **5**, 3169-3186 (2021).
47. Liu, K., *et al.* Reducing sputter induced stress and damage for efficient perovskite/silicon tandem solar cells. *J. Mater. Chem. A* **10**, 1343-1349 (2022).
48. Li, R., *et al.* $CsPbCl_3$-cluster-widened bandgap and inhibited phase segregation in a wide-bandgap perovskite and its application to NiO_X-Based perovskite/silicon tandem solar cells. *Adv. Mater.* **34**, 2201451 (2022).
49. Zheng, X., *et al.* Photoactivated p-doping of organic interlayer enables efficient perovskite/silicon tandem solar cells. *ACS Energy Lett.* **7**, 1987-1993 (2022).
50. Wu, Y., *et al.* 27.6% Perovskite/c‐Si tandem solar cells using industrial fabricated TOPCon device. *Adv. Energy Mater.* **12**, 2200821 (2022).
51. Liu, J., *et al.* Efficient and stable perovskite-silicon tandem solar cells through contact displacement by MgF_X. *Science* **377**, 302-306 (2022).
52. Ren, N., *et al.* 50°C low-temperature ALD SnO_2 driven by H_2O_2 for efficient perovskite and perovskite/silicon tandem solar cells. *Appl. Phys. Lett.* **121**, 033502 (2022).
53. Yang, G., *et al.* Defect engineering in wide-bandgap perovskites for efficient perovskite–silicon tandem solar cells. *Nat. Photonics* **16**, 588-594 (2022).
54. Ji, S. G., *et al.* Stable pure-iodide wide-band-gap perovskites for efficient Si tandem cells via kinetically controlled phase evolution. *Joule* **6**, 2390-2405 (2022).
55. Saliba, M., *et al.* Incorporation of rubidium cations into perovskite solar cells improves photovoltaic performance. *Science* **354**, 206-209 (2016).

56. Dagar, J., *et al.* Compositional and interfacial engineering yield high-performance and stable p-i-n perovskite solar cells and mini-modules. *ACS Appl. Mater. Interfaces* **13**, 13022-13033 (2021).
57. Liu, X., *et al.* Highly efficient wide-band-gap perovskite solar cells fabricated by sequential deposition method. *Nano Energy* **86**, (2021).
58. Aktas, E., *et al.* Understanding the perovskite/self-assembled selective contact interface for ultra-stable and highly efficient p–i–n perovskite solar cells. *Energy Environ. Sci.* **14**, 3976-3985 (2021).
59. Yang, G., *et al.* Stable and low-photovoltage-loss perovskite solar cells by multifunctional passivation. *Nat. Photonics* **15**, 681-689 (2021).
60. Li, Y., *et al.* Homologous bromides treatment for improving the open-circuit voltage of perovskite solar cells. *Adv. Mater.* **34**, 2106280 (2022).
61. Zheng, Y., *et al.* Downward homogenized crystallization for inverted wide-bandgap mixed-halide perovskite solar cells with 21% efficiency and suppressed photo-induced halide segregation. *Adv. Funct. Mater.* **32**, 2200431 (2022).
62. Jiang, J., *et al.* Mixed dimensionality of 2D/3D heterojunctions for improving charge transport and long-term stability in high-efficiency 1.63 eV bandgap perovskite solar cells. *Mater. Adv.* **3**, 5786-5795 (2022).
63. Wang, C., *et al.* A universal close-space annealing strategy towards high-quality perovskite absorbers enabling efficient all-perovskite tandem solar cells. *Nat. Energy* **7**, 744-753 (2022).
64. Li, B., *et al.* Suppressing interfacial recombination with a strong-interaction surface modulator for efficient inverted perovskite solar cells. *Adv. Energy Mater.* **12**, 2202868 (2022).
65. Almora, O., *et al.* Device performance of emerging photovoltaic materials (Version 3). *Adv. Energy Mater.* **13**, 2203313 (2023).
66. Castriotta, L. A., *et al.* A universal multi-additive strategy to enhance efficiency and stability in inverted perovskite solar cells. *Nano Energy* **109**, 108268 (2023).
