# Supplementary material for: Long-chain anionic surfactants enabling stable perovskite/silicon tandems with greatly suppressed stress corrosion
Source: Nat Commun. 2023 Apr 15;14:2166. doi: 10.1038/s41467-023-37877-z (PMC10105702; doi:10.1038/s41467-023-37877-z)
Supplement: Supplementary file 3 — Solar Cells Reporting Summary [file 41467_2023_37877_MOESM3_ESM.pdf]

nature research

Corresponding author(s): Jichun Ye

Solar Cells Reporting Summary

Nature Research wishes to improve the reproducibility of the work that we publish. This form is intended for publication with all accepted papers reporting the characterization of photovoltaic devices and provides structure for consistency and transparency in reporting. Some list items might not apply to an individual manuscript, but all fields must be completed for clarity.

For further information on Nature Research policies, including our data availability policy, see Authors & Referees.

Experimental design

Please check: are the following details reported in the manuscript?

1. Dimensions

Item	Yes/No	Details
Area of the tested solar cells	☒ Yes ☐ No	The active areas are 0.124 cm2 and 0.949 cm2 for small-size perovskite devices and tandem devices, respectively (Methods and Supplementary Fig. 53).
Method used to determine the device area	☒ Yes ☐ No	The device area was defined by an aperture mask (Methods).

2. Current-voltage characterization

Item	Yes/No	Details
Current density-voltage (J-V) plots in both forward and backward direction	☒ Yes ☐ No	The J–V plots for the hysteresis study were obtained from scans in both forward and backward directions unless specified otherwise. The information can be found in the Methods and the plots are showed in Fig. 4b and Supplementary Fig. 49.
Voltage scan conditions *For instance: scan direction, speed, dwell times*	☒ Yes ☐ No	All devices were measured with a scan rate of 100 mV/s with the delay time of 10 ms (Methods).
Test environment *For instance: characterization temperature, in air or in glove box*	☒ Yes ☐ No	Our devices were characterized at room temperature (ca. 25 Celsius degree) in air.
Protocol for preconditioning of the device before its characterization	☒ Yes ☐ No	No preconditioning protocol was used before the characterization. The information is stated in "Device characterization" part in "Methods".
Stability of the J-V characteristic *Verified with time evolution of the maximum power point or with the photocurrent at maximum power point; see ref. 7 for details.*	☒ Yes ☐ No	Stabilized photocurrent output by holding the voltage at the maximum power point (Supplementary Figs. 30 and 51). The stability test information can be found in the "Stability testing" part in "Methods".

3. Hysteresis or any other unusual behaviour

Item	Yes/No	Details
Description of the unusual behaviour observed during the characterization	☒ Yes ☐ No	No J–V hysteresis or any other unusual behaviour was observed and the related comments were mentioned in manuscript.
Related experimental data	☒ Yes ☐ No	We show the J–V plots under different directions in Fig. 4b and Supplementary Fig. 49.

4. Efficiency

Item	Yes/No	Details
External quantum efficiency (EQE) or incident photons to current efficiency (IPCE)	☒ Yes ☐ No	The EQE spectrum can be found in Supplementary Figs. 31 and 50.
A comparison between the integrated response under the standard reference spectrum and the response measure under the simulator	☒ Yes ☐ No	The integrated Jsc from EQE spectrum is agreed well (less than 5% mismatch) with J–V measurement. The comparison is represented in Fig. 4b and Supplementary Figs. 31.
For tandem solar cells, the bias illumination and bias voltage used for each subcell	☒ Yes ☐ No	"Device characterization" part in "Methods".

5. Calibration

Item	Yes/No	Details
Light source and reference cell or sensor used for the characterization	☒ Yes ☐ No	A solar simulator (EMS–35AAA, Ushio Spax Inc.) based on the Ushio Xe short arc lamp 500 was used to simulate sunlight irradiation of 1 sun (AM1.5G; 100 mW·cm–2). The solar simulator illumination intensity was calibrated using a KG5 reference Si-cell (Enlitech). The information can be found in the "Device characterization" part in "Methods".

Confirmation that the reference cell was calibrated and certified	☒ Yes ☐ No	The information can be found in the "Device characterization" part in "Methods".
Calculation of spectral mismatch between the reference cell and the devices under test	☒ Yes ☐ No	Spectral mismatch factor of 1 was used for all J–V measurements.

6. Mask/aperture

Size of the mask/aperture used during testing	☒ Yes ☐ No	The area of aperture mask is 0.124 cm2 (Methods).
Variation of the measured short-circuit current density with the mask/aperture area	☒ Yes ☐ No	The aperture area is fixed for each device.

7. Performance certification

Identity of the independent certification laboratory that confirmed the photovoltaic performance	☒ Yes ☐ No	We sent our device to Shanghai Institute of Microsystem and Information Technology (SIMIT) for J-V characterization according to their standard protocol (IEC60904-1 2006). The information can be found in Supplementary Fig. 53.
A copy of any certificate(s) *Provide in Supplementary Information*	☒ Yes ☐ No	SIMIT provided J-V results according to their standard protocol (IEC60904-1 2006). The information can be found in Supplementary Fig. 53.

8. Statistics

Number of solar cells tested	☒ Yes ☐ No	12 devices are provided for statistical analysis of the photovoltaic parameters (Supplementary Figs. 32, 33 and 52) and 6 devices are provided for ISOS-L2 aging test (Fig 4e,g and Supplementary Figs. 44 and 54).
Statistical analysis of the device performance	☒ Yes ☐ No	Supplementary Figs. 32, 33 and 52.

9. Long-term stability analysis

Type of analysis, bias conditions and environmental conditions *For instance: illumination type, temperature, atmosphere humidity, encapsulation method, preconditioning temperature*	☒ Yes ☐ No	The unencapsulated devices were aged using a xenon lamp with 100 mW·cm–2 irradiance at the regular air ambient environment (RH = 40-70%, T = 20-35 °C) without preconditioning. The information on the variety of aging conditions used for testing long-term stability is present throughout the manuscript and in the "Methods" section under the "Stability Testing" subsection.
